# Proton Beam Therapy for Early Breast Cancer: A Systematic Review and Meta-analysis of Clinical Outcomes

**DOI:** 10.1016/j.ijrobp.2023.02.023

**Published:** 2023-03-02

**Authors:** Francesca Holt, Jake Probert, Sarah Darby, Joanne S Haviland, Charlotte E Coles, Anna M Kirby, Zulian Liu, David Dodwell, Georgios Ntentas, Frances Duane, Carolyn Taylor

**Affiliations:** 1 Nuffield Department of Population Health, University of Oxford, UK; 2 Centre for Evaluation and Methods, Wolfson Institute of Population Health, Queen Mary University of London, UK; 3 Department of Oncology, University of Cambridge, UK; 4 Royal Marsden NHS Foundation Trust and Institute of Cancer Research, UK; 5 Department of Medical Physics, Guy’s & St Thomas’ NHS Foundation Trust, UK; 6 St. Luke’s Radiation Oncology Network and Trinity St. James's Cancer Institute, Ireland

**Keywords:** Breast cancer, Proton beam therapy, Radiotherapy, Breast reconstruction, Radiation dermatitis

## Abstract

**Background:**

Adjuvant proton beam therapy (PBT) is increasingly available to patients with breast cancer. It achieves better planned dose distributions than standard photon radiotherapy and therefore may reduce the risks. However, clinical evidence is lacking.

**Methods:**

A systematic review of clinical outcomes from studies of adjuvant PBT for early breast cancer published 2000-2022 was undertaken. Early breast cancer was defined as when all detected invasive cancer cells are in the breast or nearby lymph nodes and can be removed surgically. Adverse outcomes were summarized quantitatively, and the prevalence of the most common ones estimated using meta-analysis.

**Results:**

Thirty-two studies (1452 patients) reported clinical outcomes after adjuvant PBT for early breast cancer. Median follow up ranged from 2-59 months. There were no published randomized trials comparing PBT with photon radiotherapy. Scattering PBT was delivered in seven studies (258 patients) starting 2003-2015 and scanning PBT in 22 studies (1041 patients) starting 2000-2019. Two studies (123 patients) starting 2011 used both PBT types. For one study (30 patients) PBT type was unspecified.

Adverse events were less severe after scanning than after scattering PBT. They also varied by clinical target. For partial breast PBT, 498 adverse events were reported (8 studies, 358 patients). None were categorized as severe after scanning PBT. For whole breast or chest wall +/- regional lymph nodes PBT, 1344 adverse events were reported (19 studies, 933 patients). After scanning PBT, 4% (44/1026) of events were severe. The most prevalent severe outcome after scanning PBT was dermatitis which occurred in 5.7% (95% CI 4.2-7.6) of patients. Other severe adverse outcomes included infection, pain and pneumonitis (each ≤1%). Of the 141 reconstruction events reported (13 studies, 459 patients), the most prevalent after scanning PBT was prosthetic implant removal (34/181, 19%).

**Conclusions:**

This is a quantitative summary of all published clinical outcomes after adjuvant PBT for early breast cancer. Ongoing randomized trials will provide information on its longer-term safety compared with standard photon radiotherapy.

## Introduction

Adjuvant photon radiotherapy is given to around two thirds of women with breast cancer. ^1^ It reduces the risk of breast cancer death when targeted at the whole breast after breast conserving surgery, or the chest wall after mastectomy in lymph node positive cancer. ^2,3^ Inclusion of the regional lymph nodes further improves survival. ^4^ However, when irradiating these clinical targets, incidental irradiation of the nearby heart, lungs and esophagus can increase the risks of death from heart disease, lung and oesophageal cancer respectively. ^5–7^

During the past few decades, advances in breast cancer photon radiotherapy have reduced incidental irradiation of the surrounding organs and homogenized dose to clinical targets, likely contributing to improved clinical outcomes. ^8^ For the majority of women receiving modern photon radiotherapy who do not smoke, the absolute mortality risks are now estimated to be less than 1%.^6^ However, for some women, the risks of heart disease and second cancers may be higher. For example, it may be more difficult to spare nearby organs from radiation when treating patients with left-sided breast cancers, those requiring internal mammary lymph node irradiation, and those with unfavorable cardiothoracic anatomy. ^9–11^ Absolute radiation-risks of heart disease and lung cancer may also be higher for women who are long-term continuing smokers or have pre-existing heart disease. ^5,6^ In women with one or more of these risk factors, there may be a role for proton beam therapy (PBT). ^9–11^

Adjuvant PBT is being considered as an alternative to standard photon radiotherapy because it may further reduce radiation-related risks for patients with early breast cancer. ^5–7,12–14^ Dosimetry studies have demonstrated that when irradiating the breast or chest wall +/- regional lymph nodes PBT can achieve lower doses to the heart, lungs and esophagus compared with photon radiotherapy without compromising coverage of the clinical target. ^9–11^

The availability of PBT for breast cancer has increased over recent decades, and the technology has advanced. Since 1990 around 95 PBT centers have opened for clinical use worldwide including 41 in the USA and 30 in Europe. ^15^ Early PBT was delivered using passive scattering which spreads a narrow proton beam so that it is wide enough to cover the clinical target. By 2014 most centers were delivering PBT via uniform scanning or pencil beam scanning both of which use magnetic fields to steer narrow proton beams across the clinic target, enabling better sparing of normal tissues. ^16^ In particular better skin-sparing is possible with pencil beam scanning technology because of its enhanced dose modification capabilities.

Although PBT for breast cancer is increasingly available and the technology and techniques to deliver it are improving, its place as a cost-effective, clinically-beneficial alternative to photon radiotherapy has not yet been proven. Lack of clinical evidence is the main reason that the majority of European PBT centers are not using protons to treat patients with breast cancer routinely. ^17^

Synthesis of all available knowledge of clinical outcomes after the most advanced PBT techniques is needed to improve our knowledge of its clinical benefits and risks. This systematic review aims to summarize and quantify all clinical outcomes after adjuvant PBT for early breast cancer published before September 2022 to inform clinicians and patients, and to guide the design of future clinical trials. We also summarize ongoing studies of PBT in early breast cancer.

## Material and Methods

A systematic review was performed in accordance with the Preferred Reporting Items for Systematic Review and Meta-Analyses guidelines. ^18^ Search methods, inclusion criteria, and data abstraction were specified in the study protocol registered on PROSPERO and are described below. ^19^

### Study eligibility

Two groups of studies were included in the review. In the first group, eligible studies were those that reported clinical outcomes in patients who received adjuvant PBT for early breast cancer after curative intent surgery and were published in full text or abstract form between 1^st^ January 2000 and 1^st^ September 2022 (Text A1a, Figure A1a). Breast cancer was defined as early when all detected invasive cancer cells were in the breast or regional lymph nodes and could be removed surgically. Patients of any age or sex were included. Studies providing only the outcomes of clinical investigations (e.g. echocardiogram findings) rather than outcomes from clinical history or examination were excluded because such surrogates for clinical outcomes have yet to be validated for clinical use in this context. Studies were excluded if they were case reports or if clinical outcome data could not be extracted.

The second group of eligible studies included all ongoing clinical studies of adjuvant PBT in women with early breast cancer after curative intent surgery (Text A1b, Figure A1b). These studies were recruiting patients, or had completed recruitment and were in follow-up, but had not yet published their results.

### Study Identification

Publications reporting clinical outcomes after adjuvant PBT for early breast cancer were identified by searching Ovid MEDLINE® and Embase using the following terms: breast cancer NOT advanced/metastatic/inoperable, AND proton radiotherapy (Text A1a). Full text publications and abstracts were included to capture the clinical outcomes from as many patients as possible.

Ongoing clinical studies of PBT in breast cancer were identified from the ClinicalTrials.gov website, the World Health Organisation International Clinical Trials Registry Platform, and the Cochrane Central Register of Controlled Trials using the search terms ‘breast cancer’ AND ‘proton’ (Text A1b).^20–22^

### Data abstraction

From eligible published studies reporting clinical outcomes, the following information was abstracted: study author, years of study and location, study design, number of patients, number of breast cancers, details of primary and reconstructive breast cancer surgery, details of PBT beam delivery type and technique, PBT dose (Gy) and fractionation, median follow-up, breast cancer outcomes, number, type and severity of adverse clinical outcomes, and the clinical definitions and severity grading systems used. Where the PBT delivery type or clinical target were unspecified, the corresponding authors of the studies were contacted for further information.

To prevent counting of individual patient clinical outcomes more than once, studies analysing multi-institutional registry data on clinical outcomes after PBT for early breast cancer were excluded from further analysis (Figure A1a, Table A1). The remaining publications were grouped into the individual studies they were reporting on using trial IDs. Publications without trial ID were grouped as an individual study if the abstracted information suggested that the publications were reporting upon the same patients. Each of the resulting studies was assigned one main study publication from which reported clinical outcomes contributed to data analysis. The main study publication was selected as the most recent full text publication presenting clinical outcomes, or the most recent abstract where a full text publication was not available.

From eligible ongoing clinical studies, the following information was abstracted: study design, study location and years, estimated number of patients, details of planned PBT intervention, and planned study outcomes.

### Data analysis

Individual published studies were categorized by PBT clinical target: partial breast, whole breast or chest wall +/- regional lymph nodes, and reconstructed breast +/- regional lymph nodes. It was not possible to separate clinical targets further as this level of detail was not published in the majority of studies. Within these three groups, studies were categorized by PBT delivery type: scattering, scattering or scanning, scanning, and unspecified. Scattering PBT included both passive and double scattering. Scanning PBT included both uniform scanning and pencil beam scanning.

Adverse clinical outcomes reported in individual published studies were categorized as mild (or grade 1), moderate (or grade 2), severe (or grade 3), or unspecified, using the study’s specified grading system (for definitions of dermatitis see Table A2). For each study, the number of patients reported to be affected by, and the number assessed for each grade of each type of reported adverse outcome was counted. Where a study reported the number of events for an outcome at more than one point in time, then only the time point with the highest number of severe events was counted for that outcome in that study, although different time points may have been selected for different outcomes.

Within each category of PBT clinical target and PBT delivery type, adverse outcomes were ranked in descending order of severity using the percentage of patients affected. Where the percentages of patients affected were the same, adverse outcomes were arranged in descending order of the number of patients affected.

The highest number of severe adverse events was reported for dermatitis in women who received PBT to the whole breast or chest wall +/- regional lymph nodes. Fisher’s exact test was used to evaluate the proportions of severe dermatitis reported between studies using scattering and scanning PBT. Then, because the outcomes after scanning PBT are most relevant to current clinical practice, we quantified the prevalence of dermatitis after scanning PBT for early breast cancer to the whole breast or chest wall +/- regional lymph nodes using meta-analysis of proportions. ^23^ Confidence intervals for the observed prevalence in individual studies were calculated using the exact binomial method. Between-study heterogeneity was assessed by p-value from Wald-type tests and, as heterogeneity was present in some cases, pooled estimates were obtained using generalized linear mixed models with a logit link function (using R package *metafor*). Random effects models were used since such analyses are often highly heterogeneous. Individual study weights were derived from the likelihood contribution of each individual study. The overall prevalence estimates presented may be interpreted as the median prevalence from multiple studies.

For breast cancer outcomes, the number of patients affected by, and assessed for, locoregional recurrence, distant recurrence, breast cancer death and death from any cause were counted. Analyses of these data were not possible as information regarding the time point of individual breast cancer outcomes was missing from most publications.

Ongoing clinical studies were categorized by study design into randomized and non-randomized studies, then by PBT clinical target. Summarized data were tabulated.

## Results

### Published studies

Thirty-two individual published studies (1452 patients) reporting clinical outcomes after adjuvant PBT for early breast cancer were identified. Study start dates ranged from 2003 to 2019 (Figure A1a, Table 1). There were no randomized studies comparing PBT with photon radiotherapy. Clinical outcomes were published in full text for 21 studies (775 patients) and in abstracts for 11 studies (677 patients).

**Figure 1:**
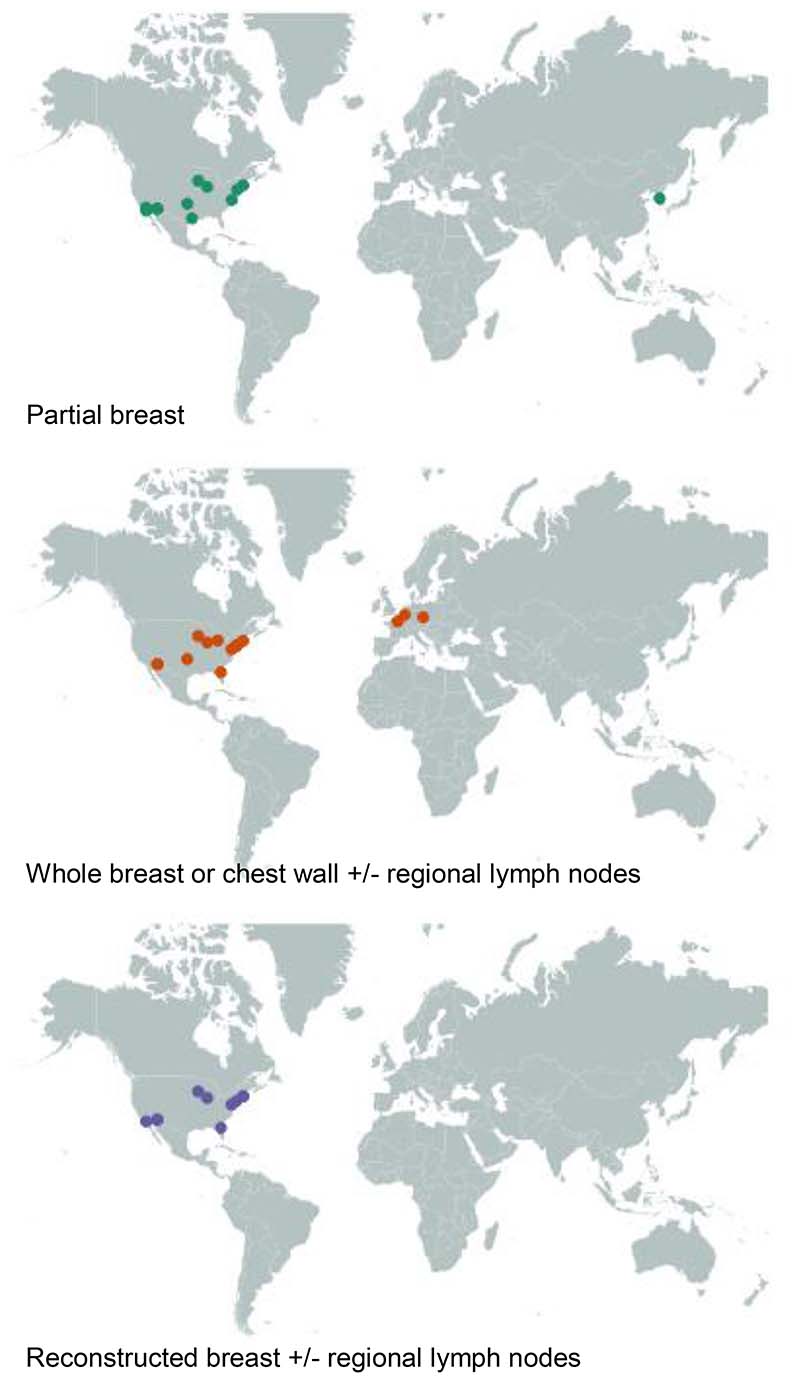
Global distribution of centers reporting clinical outcomes after proton beam therapy for early breast cancer in studies published 2000-2022 according to clinical target.

The majority of studies (28/32 studies, 1359 patients) were conducted in the USA (Table 1, Figure 1). One center in the Republic of Korea (30 patients) reported clinical outcomes after partial breast PBT and one center located in each of the Czech Republic (42 patients), the Netherlands (20 patients), and France (one patient) reported clinical outcomes after PBT to the whole breast or chest wall +/- regional lymph nodes. No center outside the USA reported clinical outcomes after PBT to the reconstructed breast.

PBT clinical targets under investigation have changed with time. The partial breast was targeted in eight studies (358 patients) published since 2006. Reported study start dates ranged from 2003 to 2015 (Table 1). The whole breast or chest wall +/- regional lymph nodes has been targeted in 19 studies (933 patients) published since 2016. Reported study start dates ranged from 2011 to 2019 (Table 1). Reconstruction outcomes after PBT to the reconstructed breast +/- regional lymph nodes were reported in 13 studies (459 patients) published since 2016. Reported study start dates ranged from 2000 to 2016 (Table 1).

PBT delivery types have also changed with time. PBT was delivered using a scattered beam in seven studies (258 patients) published since 2006. Reported start dates ranged from 2003 to 2015. Scanning PBT was used in 22 studies (1041 patients) published since 2017. Reported start dates ranged from 2000 to 2019. Two studies (123 patients) both starting in 2011 used both PBT types. One study (30 patients) did not specify the type of PBT used. (Table 1)

Adverse outcomes varied by clinical target and PBT delivery type (Tables 2 and 3).

#### Partial breast PBT: adverse outcomes

PBT dose and fractionation varied according to clinical target. All eight studies of partial breast PBT investigated accelerated partial breast irradiation (Table 1). Six of these used total doses ranging from 32 to 40 Gy in 3.4 to 4 Gy fractions given across 4 and 14 days. The other two studies used higher doses per fraction: 21.9 Gy in 3 daily fractions (7.3 Gy per fraction), and 30 Gy in 5 daily fractions (6 Gy per fraction).

Scattering PBT was received by 53% (189/358) of patients and scanning PBT by 39% (139/358). For 8% (30/358) of patients PBT type was unspecified (Table 1). Median follow-up of patients across studies ranged from 12 to 59 months.

**Figure 2:**
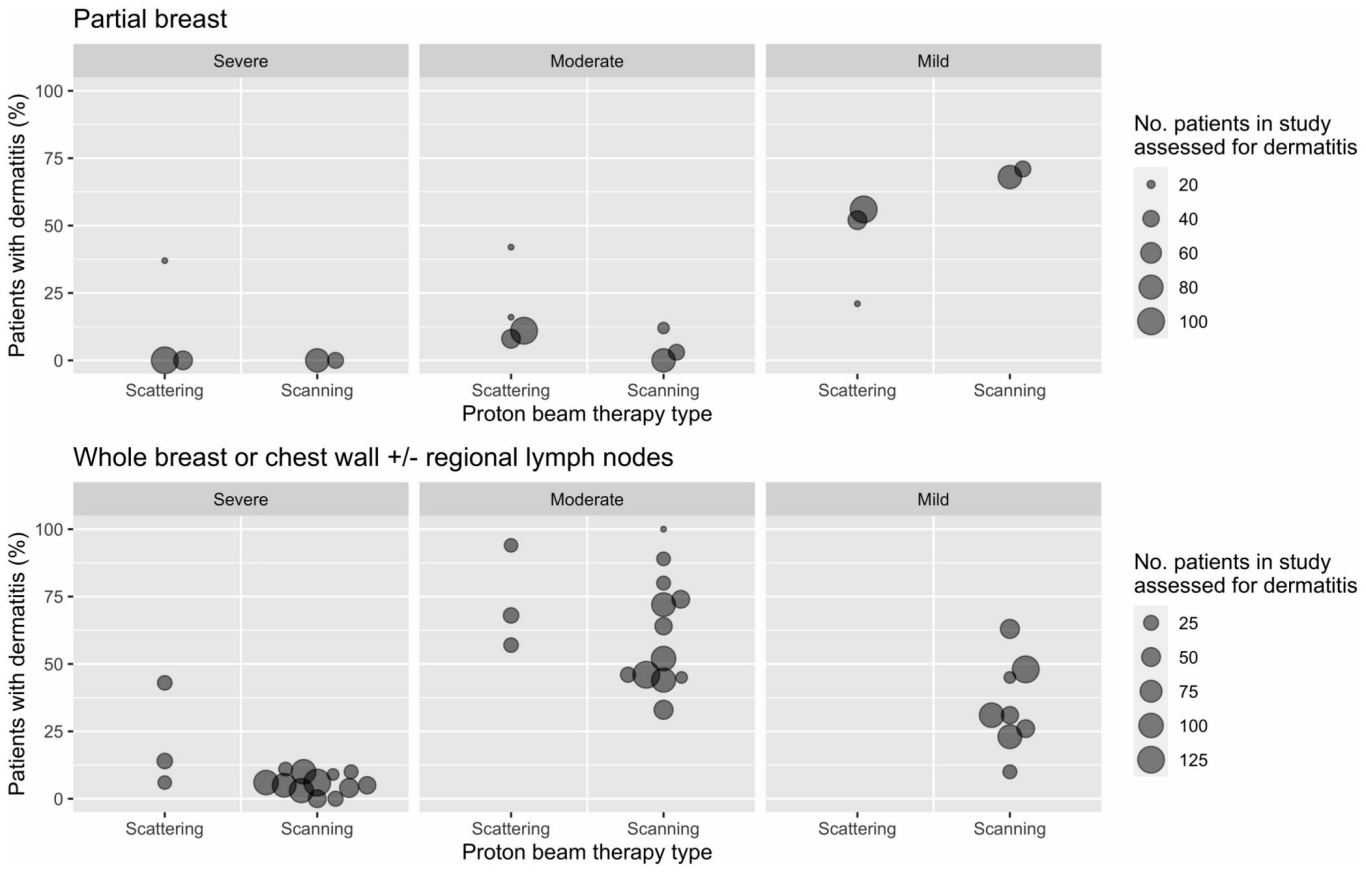
Percentage of patients with dermatitis after proton beam therapy for breast cancer in studies published 2000-2022, according to clinical target and proton beam therapy type. For each individual study reporting dermatitis, the percentage of patients with each grade of dermatitis was plotted. See Table A4 for patients with unspecified grade of dermatitis, patients who received scanning or scattering proton beam therapy, and unspecified proton beam type. Abbreviations: No. = number, PBT = proton beam therapy

For partial breast PBT, 498 adverse events were reported. These spanned 14 types of adverse outcomes, all of which related to effects on breast skin and soft tissue (Table 2a). These adverse outcomes were reported using Common Terminology Criteria for Adverse Events (CTCAE) versions 2.0 or 4.0 in four studies, categories of ‘mild’, ‘moderate’ and ‘severe’ in two studies, and the Radiation Therapy Oncology Group (RTOG)/European Organisation for Research and Treatment of Cancer (EORTC) grading system in one study (Tables A4 and A5). For the remaining study, the system used to grade adverse outcomes was unspecified. The timing of adverse outcomes was between 0 and 84 months where specified. Information on timing was missing in 23% (12/52) of instances (Tables A4 and A5). Of the 498 adverse events reported, 2% (8/498) were graded as severe, 13% (67/498) as moderate, and 79% (393/498) as mild. Grade was unspecified in 6% (30/498) of events. The only outcome with severe events was dermatitis. Severe dermatitis events were all graded using a system that enabled classification as ‘mild’, ‘moderate’ or ‘severe’, and were reported at two months post PBT. There were more reports (p=0.02) of severe dermatitis in patients assessed after scattering PBT (4%, 7/169) than after scanning PBT (0%, 0/114). There was one event (3%, 1/30) after PBT of unspecified type (Table 2a, Figure 2, Table A4).

#### Whole breast or chest wall +/- regional lymph nodes PBT: adverse outcomes

Studies of whole breast or chest wall +/- regional lymph nodes PBT reported outcomes after 45 to 50 Gy total dose in 1.8 or 2 Gy fractions in 15/19 studies (Table 1). The other 4/19 studies included patients receiving hypofractionated regimens of 40 to 43 Gy in 15 or 16 fractions.

Scattering PBT was received by 7% (69/933) of patients and scanning PBT by 85% (795/933). One study (69/933, 7%) used either scattering or scanning (Table 1). Median follow-up ranged from 2 to 55 months.

For whole breast or chest wall +/- regional lymph nodes PBT, 1344 adverse events were reported. These spanned 15 types of adverse outcomes relating to effects on breast or chest wall skin and soft tissue, esophagus, lungs, ribs, lymphatics, and brachial plexus (Table 2b). These adverse outcomes were reported using Common Terminology Criteria for Adverse events (versions 3.0, 4.0 or 5.0) in 14 studies, and RTOG/EORTC in one study (Tables A4 and A6). For the remaining four studies, the system used to grade adverse outcomes was unspecified. Where specified, the adverse outcomes were assessed between 0 and 39 months after PBT, but information on the timing was missing in 58% (59/104) instances (Tables A4 and A6). Of the 1344 adverse events, 5% (65/1344) were graded as severe, 45% (604/1344) as moderate, and 47% (629/1344) as mild. For 3% (46/1344) of adverse events the grade was unspecified. The most prevalent severe outcome was dermatitis. Outcomes of severe dermatitis were all graded using CTCAE versions 3.0-5.0 and were reported within 6 months post PBT where specified. The grading system was unspecified in 13% (2/16) of studies reporting on severe dermatitis, and the timing unspecified in 69% (11/16) of studies. Severe dermatitis was reported in 22% (15/69) of assessed patients after scattering PBT compared with only 6% (41/723) after scanning PBT (p<0.001). Two reports of severe dermatitis (3%, 2/69) occurred in a study of scattering or scanning PBT (Table 2b, Figure 2, Table A4). Other severe adverse outcomes were infection, pain and pneumonitis. Outcomes of severe infection were all graded using CTCAE versions 4.0-5.0, and were reported between 4 to 32 months where specified. The timing was unspecified in 50% (2/4) of studies reporting upon severe infection. Severe infection was reported in 17% of patients (3/18) after scattering PBT, 1% (1/69) after scattering or scanning, and in 1% (1/137) after scanning PBT. Severe pain was reported in one patient (1/246, <1%), as was pneumonitis (1/257, <1%). Both of these outcomes were graded using CTCAE v4.0 and occurred after scanning PBT (Tables 2b and A6). The timing of the outcome of severe pain was unspecified. The outcome of pneumonitis was reported at 12 months.

**Figure 3:**
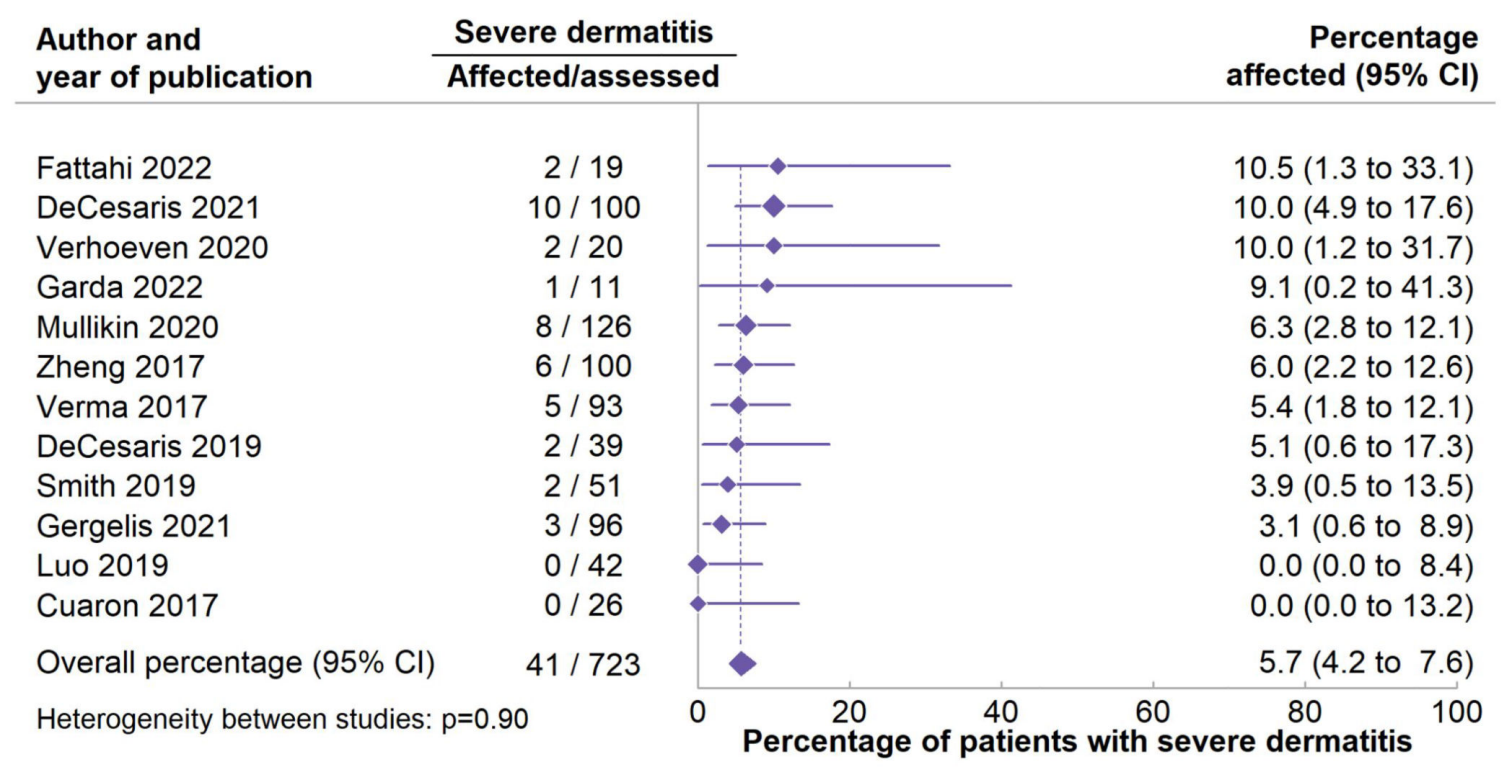
Meta-analysis of the percentage of patients with severe dermatitis after whole breast or chest wall +/- regional lymph nodes scanning proton beam therapy in studies published 2000-2022. See Figure A2 for meta-analyses of the percentage of patients with moderate and mild dermatitis.

Meta-analysis of the percentage of patients affected by severe dermatitis after scanning PBT to the whole breast or chest wall +/- regional lymph nodes included 41 events from 723 patients in 12 studies (Figure 3). Although the estimated number of patients with severe dermatitis per 100 patients assessed in different studies varied from 0-10.5 in different studies, there was no significant heterogeneity between studies (p=0.90). Overall, severe dermatitis occurred in 5.7 (95% CI 4.2-7.6) per 100 patients.

Meta-analysis of the percentage of patients affected by moderate dermatitis included 343 events from 624 patients in 12 studies (Figure A2). The estimated number of patients with moderate dermatitis per 100 patients assessed in different studies varied from 33.3 to 100.0. There was significant heterogeneity between the studies (p<0.001) and the overall estimate, allowing for heterogeneity was 59.3 (48.5-69.3) per 100 patients. Meta-analysis of the percentage of patients affected by mild dermatitis (Figure A2) included 174 events from 482 patients in eight studies. The estimated number of patients with mild dermatitis per 100 patients assessed in different studies varied from 10.0 to 62.7. There was significant heterogeneity between the studies (<0.001) and the overall estimate, allowing for heterogeneity was 33.7 (23.8-45.3) per 100 patients.

#### Reconstructed breast +/- regional lymph nodes PBT: adverse reconstruction outcomes

There were 13 studies that reported reconstruction outcomes after reconstructed breast +/- regional lymph node PBT (Table 3). Five of these were studies that only included patients with breast reconstructions. The remaining eight were studies that included some patients with breast reconstructions and some without (Table 1). Of the 11/13 studies that specified the PBT total dose and fractionation, 10 reported outcomes after 45 to 50 Gy total dose given in 1.8 or 2 Gy fractions. One study reported outcomes after 40 Gy in 10 fractions.

Scattering PBT was delivered to 1% of irradiated breast reconstructions (4/459), scanning or scattering to 23% (107/459), and scanning PBT to 76% (348/459) (Table 3). Median follow-up of patients across studies ranged from 5 to 55 months.

For reconstructed breast PBT, 141 adverse reconstruction events were reported. These included infection in 11% (22/200) of assessed patients, and capsular contraction in 18% (47/258). For infection, the severity was unspecified in 60% (3/5) of studies reporting this outcome, and the timing unspecified in all studies. For capsular contraction, the grade and timing were unspecified in 85% (6/7) of studies reporting this outcome. Some adverse events required surgical intervention. For implant reconstructions, 12% (15/130) required surgical revision and 19% (55/286) required removal. For autologous reconstructions, 3% (1/32) required surgical revision and 50% (1/2) required surgical replacement (Table 3). The timings of these outcomes were unspecified.

#### Breast cancer outcomes

In total 19 locoregional recurrences were reported from 867 patients in 19 studies, 53 distant recurrences were reported from 811 patients in 16 studies. There were nine breast cancer deaths reported from 383 patients assessed in nine studies, and 22 deaths from any cause reported from 499 patients assessed in 11 studies. Further analysis was not performed because most studies did not provide information on the timings of these events.

### Ongoing studies

Twenty-two ongoing studies (9791 patients) collecting clinical outcomes after PBT for early breast cancer were identified including six randomized (3298 patients) and 14 non-randomized studies (6493 patients) (Figure A1b, Tables A7a and A7b). One randomized study (NCT02603341) has two associated ancillary observational studies (NCT04361240 and NCT03270072).

All randomized studies are investigating PBT to the whole breast or chest wall +/- regional lymph nodes. Five are comparing PBT with photon radiotherapy, and one is comparing standard fractionation PBT with hypofractionated PBT. Three of the six randomized studies are located in the USA, one in Denmark, one in the UK, and one in Thailand.

Of the 14 non-randomized studies, six studies are investigating PBT to the partial breast (765 patients) and three are investigating PBT to the whole breast or chest wall +/- regional lymph nodes (285 patients). All nine of these studies are located in the USA. The PBT clinical target could not be ascertained in the other five non-randomized studies (5443 patients). Two of these are located in Japan, one in the USA, one in China, and one in India.

For all ongoing studies of PBT to the whole breast chest wall +/- regional lymph nodes, planned primary outcomes are assessing adverse outcomes, with measures of efficacy as secondary outcomes. For ongoing studies of partial breast PBT, planned primary outcomes measures include efficacy (two studies), cosmesis (two studies), and adverse outcomes (two studies).

## Discussion

This systematic review provides a quantitative summary of reported adverse clinical outcomes after different types of PBT for early breast cancer using data published during 2000-2022. Most studies had median follow-up of less than two years, with little data on outcomes beyond three years. The commonest reported adverse outcome overall was dermatitis. Adverse outcomes were less severe after PBT using the latest scanning technology than after PBT using the older scattering technology, suggesting that advances in PBT technology have improved the clinical safety of PBT for early breast cancer within the first three years. Adverse outcomes were also less severe after PBT to the partial breast, than after PBT to the whole breast or chest wall +/- regional lymph nodes, as expected given that the partial breast is a smaller and more superficial target volume.

The main strength of this review is that it provides quantitative estimates of the prevalence of adverse outcomes after PBT for early breast cancer using published data. To the best of our knowledge it is also the first systematic review to summarize reconstruction outcomes after PBT for early breast cancer. Our searches were systematic with inclusive search terms, so we are likely to have identified and assessed all relevant studies. We included data from all eligible studies published as either abstracts or full texts since the year 2000, making it the largest review of its kind. Quantitative synthesis of available data was made possible by categorizing adverse events by severity, and extracting numbers of patients with each type of adverse outcome and the numbers assessed from each study at the time point at which the highest number of patients were reported with severe adverse outcomes.

There are three other published systematic reviews summarizing clinical outcomes after adjuvant PBT for early breast cancer. ^24–26^ These were published during 2016-2022. Alterio et al. 2022 summarized clinical outcomes from five studies (326 patients) investigating adjuvant PBT to the partial breast.^24^ Kammerer et al. 2018 summarized clinical outcomes from 3 studies (135 patients) of adjuvant PBT to the whole breast or chest wall +/- regional lymph nodes.^25^ Verma et al. 2016 summarized clinical outcomes from 4 studies (102 patients) of adjuvant PBT to the whole breast or chest wall +/- regional lymph nodes and 5 studies (197 patients) of adjuvant PBT to the partial breast.^26^ These reviews included fewer studies and reported fewer clinical outcomes than our review and all presented qualitative summaries of the data.

A limitation of our study is that the estimates of the prevalence of adverse outcomes provided are informed by published summary data rather than individual patient data. This means that some instances of duplication of patient outcomes may remain undetected despite careful grouping of publications into individual studies. In addition, the extent to which patients in these studies represent patients being considered for PBT today is uncertain because detailed information on characteristics of patients selected for these studies was lacking. Information on the timing of reported outcomes was often missing. Therefore it was neither possible to classify adverse outcomes into acute and late effects, nor to summarize the duration of these outcomes. These factors need to be considered when applying our overall summary estimates of prevalence of adverse effects to patients in the clinic today. For breast cancer outcomes, missing information on the timing of individual outcomes prevented further analysis of these outcomes.

For partial breast PBT most adverse outcomes were graded as mild and there were no severe adverse outcomes from scanning PBT to the partial breast. However, the main justification of the extra cost of PBT compared with standard photon radiotherapy is the predicted reduction in the risks of cardiovascular disease and second cancers. For partial breast photon radiotherapy, these risks are likely to be very small for most patients because radiation doses to the heart, lungs and esophagus are usually <1Gy. ^9–11^ Therefore it is more difficult to justify the excess costs of partial breast PBT compared with partial breast photon radiotherapy.

For PBT to the whole breast or chest wall +/- regional lymph nodes, the most frequently reported severe adverse outcome was dermatitis. This was most commonly defined as moist desquamation in areas other than skin folds and creases, or bleeding induced by minor trauma or abrasion. Although the timing of severe dermatitis was mostly unreported, where it was specified it peaked at less than six months post PBT. It occurred in around 6% of patients after scanning PBT, which is of a similar magnitude to expected rates of dermatitis using the latest hypofractionated (40Gy/15 fractions and 26Gy/5 fractions) photon radiotherapy techniques to the whole breast or chest wall. ^27^ Other severe adverse outcomes after scanning PBT included infection, pain and pneumonitis. These occurred in ≤1% of patients. This reassuring safety information supports the rationale for ongoing clinical trials of PBT to the whole breast or chest wall +/- regional lymph nodes for early breast cancer and it may inform patient consent whilst we await the results of randomized controlled trials.

For scanning PBT to the reconstructed breast, the most commonly reported adverse outcome was removal of prosthetic implant, which occurred in 19% of patients assessed. This may be higher than expected in current clinical practice using photon radiotherapy where estimates are around 9%.^28,29^ However, estimates of the prevalence of reconstruction outcomes after radiotherapy vary considerably and without information on the timing of reconstruction outcomes comparison between studies is difficult. Published data only include reconstruction outcomes of 348 breast reconstructions irradiated with scanning PBT for early breast cancer after a median follow up less than three years, so further data are needed.

Our review demonstrates the need for more data on clinical outcomes after PBT for early breast cancer. There were no published randomized controlled trials comparing PBT with photon radiotherapy. In most studies there were less than 2 years of follow up, with insufficient data to assess long-term clinical outcomes of PBT. Furthermore, most studies presented outcomes from patients irradiated in single privately-funded centers in the USA. Ongoing studies include five randomized trials that compare PBT with photon radiotherapy, and plan to recruit 3210 patients. One of these trials (98 patients) includes patients with breast reconstruction and aims to assess unplanned surgical interventions. More clinical trials of PBT in early breast cancer are being planned, and our data may guide their design.

In conclusion, PBT technology has advanced over the past two decades so that most patients with early breast cancer receiving PBT would now have it delivered using scanning technology. Published studies with few outcomes reported beyond three years suggest that the most common severe adverse outcome after scanning PBT to the whole breast or chest wall +/- regional lymph nodes is dermatitis which occurs in 6% of patients (95% CI 4-8). Other severe adverse outcomes from scanning PBT to the whole breast or chest wall +/- regional lymph nodes are much rarer, ≤1%. No severe adverse outcomes after partial breast scanning PBT have been reported. Our estimates of the prevalence of adverse outcomes after irradiation of different clinical targets may inform clinicians and patients considering PBT. However, few published data are available. Over the next 15 years, ongoing randomized clinical trials are likely to provide information on the longer-term safety of PBT compared with photons in patients with early breast cancer.

## Supplementary Material

Appendices

## Figures and Tables

**Table 1:** Individual studies reporting clinical outcomes after proton beam therapy for early breast cancer published 2000 - 2022 according to clinical target and proton beam therapy type

Author and year of publication*	Country	Year study started	No. patients received PBT (N=1452)	Total dose (Gy)/ No. fractions	Median follow up (months)
**Partial breast (358 patients)**					
*Scattering (189 patients)*					
Galland-Girodet 2014^10–13^^†^	USA	2003	19	32/8	–
Kozak 2006^9^^†^	USA	2004	20	32/8	12
Pasalic 2021^1–3^	USA	2010	100	34/10	24
Bush 2011^4–8^	USA	—	50	40/10	48
*Scanning (139 patients)*					
Choi 2022^16–18^	USA	2013	38	40/10	35
Giap 2017^19, 20^^‡§^	USA	2014	25	40/10	19
Mutter 2019^14, 15^	USA	2015	76	22/3	12
*Type unspecified (30 patients)*					
Chang 2013^21–22^	Republic of Korea	2007	30	30/5	59
**Whole breast or Chest wall +/- regional lymph nodes (933 patients)**					
*Scattering (69 patients)*					
Bradley 2016^26–33^^§^	USA	2012	18	50/28	20
Liang 2018^25^	USA	2012	23	50/25-28	–
Sayan 2022^23, 24^	USA	2015	28	40-50/-	27
*Scattering or scanning (69 patients)*					
Jimenez 2019^34–38^^§^	USA	2011	69	45-50/25-28	55
*Scanning (795 patients)*					
Verma 2017^43–46^^§^	USA	2011	91	50/25-28	16
Luo 2019^49–52^^§^	USA	2013	42	50/-	35
Pasztorova 2018^53^^‡^	Czech Republic	2015	42	40/15 or 50/25	7
DeCesaris 2019^54^	USA	2015	39	45-50/25-28	–
Smith 2019^47, 48^^§^	USA	2015	51	50/25	19
Gergelis 2021^42^^‡§^	USA	2015	96	50/25	–
Garda 2022^60^	USA	2015	11	50/25	32
DeCesaris 2021^41^^‡^	USA	2016	100	50/25-28	15
Fattahi 2022^58, 59^	USA	2016	19	50/25	24
Loap 2021^61^	France	2019	1	50/25	6
Zheng 2017^40^^‡¦^	USA	–	100	45-50/25-28	–
Cuaron 2017^56^^‡^	USA	–	26	49/-	15
Mullikin 2020^39^^‡§^	USA	–	126	50/25	18
Verhoeven 2020^57^^‡^	Netherlands	–	20	40/15	–
Salari 2021^55^^‡^	USA	–	31	43/16 or 50/25	2
**Reconstructed breast only +/- regional lymph nodes (161 patients)**					
*Scattering or scanning (54 patients)*					
Jimenez 2020^62^^‡^	USA	2011	54	–	–
*Scanning (107 patients)*					
Naoum 2022^65–66^	USA	2000	17	–	–
Anderson 2021^67–68^	USA	2010	4	50/-	–
Nichols 2020^63^^‡^	USA	–	57	50/-	14
DeCesaris 2021^64^	USA	–	29	50/28	5

* The main study publication is referenced first, followed by other study publications (see Text A2 for references).

† Two fractions delivered daily. In all other studies where duration of treatment was reported, one fraction was delivered daily.

‡ Main study publication an abstract rather than a full text.

§ Studies included some patients who had breast reconstructions.

¦ Adverse outcomes data obtained from the Proton Collaborative Group trial REG001-09.

Abbreviations: Gy=Gray, N=total number, No.=number, PBT=proton beam therapy, USA=United States of America, "-"=not specified

**Table 2a:** Partial breast proton beam therapy: Adverse outcomes published 2000 - 2022 according to proton beam therapy type

Adverse outcome	Severe		Moderate		Mild		Unspecified	
	Affected/assessed	%	Affected/assessed	%	Affected/assessed	%	Affected/assessed	%
*Scattering (296 adverse events)*								
Dermatitis*	7/169	***4***	26/188	***14***	86/169	***51***	–	–
Atrophy	–	–	10/19	***53***	–	–	–	–
Fibrosis^†^	0/19	***0***	1/19	***5***	3/19	***16***	10/19	***53***
Fatigue	0/100	***0***	4/100	***4***	10/100	***10***	–	–
Pain^‡^	0/119	***0***	3/119	***3***	34/119	***29***	4/19	***21***
Hyperpigmentation	0/100	***0***	2/100	***2***	44/100	***44***	–	–
Infection	0/100	***0***	1/100	***1***	0/100	***0***	0/50	***0***
Breast oedema	0/119	***0***	1/119	***1***	15/119	***13***	–	–
Fat necrosis	0/163	***0***	1/163	***1***	0/163	***0***	2/69	***3***
Telangiectasia	–	–	–	–	20/150	***13***	10/39	***26***
Rib fractures	–	–	–	–	–	–	2/89	***2***
Pneumonitis^§^	0/20	***0***	0/20	***0***	0/20	***0***	0/50	***0***
Cardiac	–	–	–	–	–	–	0/50	***0***
**Subtotal**	**7/909**	***1***	**49/947**	***5***	**212/1059**	***20***	**28/385**	***7***
*Scanning (142 adverse events)*								
Dermatitis*	0/114	***0***	4/139	***3***	79/114	***69***	–	–
Fatigue	0/38	***0***	1/38	***3***	13/43	***30***	–	–
Lymphoedema	0/38	***0***	1/38	***3***	2/38	***5***	–	–
Breast oedema	0/76	***0***	0/76	***0***	17/76	***22***	–	–
Hyperpigmentation	0/72	***0***	0/72	***0***	13/72	***18***	–	–
Pain^‡^	0/114	***0***	0/114	***0***	9/114	***8***	–	–
Telangiectasia	0/110	***0***	0/110	***0***	2/110	***2***	–	–
Fibrosis^†^	0/76	***0***	0/76	***0***	1/76	***1***	–	–
Infection	0/76	***0***	0/76	***0***	0/76	***0***	–	–
Pneumonitis^§^	0/76	***0***	0/76	***0***	0/76	***0***	–	–
Rib fractures	–	–	–	–	–	–	0/76	***0***
**Subtotal**	**0/790**	***0***	**6/815**	***1***	**136/795**	***17***	**0/76**	***0***
*Type unspecified (60 adverse events)*								
Dermatitis*	1/30 ***3***	1/30	***3***	6/30	***20***	–	–
Hyperpigmentation	0/30	***0***	9/30	***30***	21/30	***70***	–	–
Fibrosis^†^	0/30	***0***	2/30	***7***	7/30	***23***	–	–
Pain^‡^	0/30	***0***	0/30	***0***	11/30	***37***	–	–
Rib fractures	–	–	–	–	–	–	2/30	***7***
**Subtotal**	**1/120**	***1***	**12/120**	***10***	**45/120**	***38***	**2/30**	***7***
**Total** *(all PBT types)*	**8/1819**	***0***	**67/1882**	**4**	**393/1974**	**20**	**30/491**	***6***

Denominators vary as not all studies assessed all grades of all adverse outcomes. See Tables A4, A5 & A6 for outcomes by study.

* Includes acute skin colour change

† Includes reports of induration.

‡ Includes reports of pain from skin, breast and unspecified pain.

§ Includes reports of cough and dyspnoea.

¦ Includes reports of dysphagia

See Table A3 for outcomes excluded from this analysis.

Abbreviations: PBT=proton beam therapy type, “-“=not specified

**Table 2b:** Whole breast or chest wall +/- regional lymph nodes proton beam therapy: Adverse outcomes published 2000 - 2022 according to proton beam therapy type

Adverse outcome	Severe		Moderate		Mild		Unspecified	
	Affected/assessed	%	Affected/assessed	%	Affected/assessed	%	Affected/assessed	%
*Scattering (104 adverse events)*								
Dermatitis	15/69	***22***	49/69 ***71***	–	–	–	–
Infection	3/18	***17***	3/18	***17***	–	–	–	–
Fatigue	0/18	***0***	6/18	***33***	–	–	–	–
Pain^‡^	0/18	***0***	9/28	***32***	–	–	1/18	***6***
Oesophagitis^¦^	0/18	***0***	6/46	***13***	–	–	–	–
Pneumonitis^§^	0/18	***0***	2/18	***11***	–	–	–	–
Lymphoedema	0/18	***0***	1/18	***6***	–	–	5/28	***18***
Atrophy	0/18	***0***	1/18	***6***	–	–	–	–
Cardiac	0/18	***0***	–	–	–	–	2/18	***11***
Rib fractures	—	*—*	–	–	–	–	1/18	***6***
**Subtotal**	**18/213**	***8***	**77/233**	***33***	**–**	**–**	**9/82**	***11***
*Scattering or scanning (214 adverse events)*								
Dermatitis	2/69	***3***	57/69	***83***	10/69	***14***	–	–
Infection	1/69	***1***	0/69	***0***	0/69	***0***	–	–
Fatigue	0/69	***0***	24/69	***35***	38/69	***55***	–	–
Oesophagitis^¦^	0/69	***0***	5/69	***7***	19/69	***28***	–	–
Pneumonitis^§^	0/69	***0***	1/69	***1***	3/69	***4***	–	–
Hyperpigmentation	–	–	–	–	36/69	***52***	–	–
Telangiectasia	–	–	–	–	11/69	***16***	–	–
Rib fractures	0/69	***0***	0/69	***0***	5/69	***7***	–	–
Lymphoedema	0/69	***0***	0/69	***0***	1/69	***1***	–	–
Atrophy	–	–	–	–	1/69	***1***	–	–
**Subtotal**	**3/483**	***1***	**87/483**	***18***	**124/690**	***18***	**–**	**–**
*Scanning (1026 adverse events)*								
Dermatitis	41/723	***6***	343/624 ***55***	174/482	***36***	5/31	***16***
Infection	1/137	***1***	2/156	***1***	0/137	***0***	7/91	***8***
Pain^‡^	1/246	***<1***	37/372	***10***	57/266	***21***	6/31	***19***
Pneumonitis^§^	1/257	***<1***	0/257	***0***	2/276	***1***	–	–
Oesophagitis^¦^	0/336	***0***	43/355	***12***	62/355	***17***	2/100	***2***
Fatigue	0/144	***0***	7/144	***5***	61/144	***42***	–	–
Decreased shoulder movement	0/11	***0***	1/30	***3***	6/30	***20***	–	–
Fibrosis^†^	0/11	***0***	1/37	***3***	9/56	***16***	–	–
Lymphoedema	0/153	***0***	3/172	***2***	21/172	***12***	3/91	***3***
Hyperpigmentation	0/176	***0***	3/218	***1***	106/244	***43***	8/19	***42***
Brachial plexopathy	0/204	***0***	0/204	***0***	3/204	***1***	–	–
Telangiectasia	0/139	***0***	0/181	***0***	3/207	***1***	0/13	***0***
Rib fractures	0/205	***0***	0/205	***0***	1/205	***0***	6/203	***3***
Cardiac	0/220	***0***	0/220	***0***	0/220	***0***	–	–
**Subtotal**	**44/2962**	***1***	**440/3175**	***14***	**505/2998**	***17***	**37/579**	***6***
**Total** *(all PBT types)*	**65/3658**	***2***	**604/3891**	***16***	**629/3688**	***17***	**46/661**	***7***

Footnotes as for Table 2a

**Table 3:** Reconstructed breast proton beam therapy: Adverse reconstruction outcomes published 2000 - 2022 according to proton beam therapy type.

Author and year of publication	Median follow up (months)	No.recon.	Adverse reconstruction outcomes											
			Infection		Capsular contraction		Prosthetic reconstruction				Autologous reconstruction			
			Revised		Removed		Revised		Replaced	
Affected/assessed	%	Affected/assessed	%	Affected/assessed	%	Affected/assessed	%	Affected/assessed	%	Affected/assessed	%
*Scattering (1 reconstruction outcome)*														
Bradley 2016*	20	4	1/4	***25***	**–**	–	–	–	–	–	–	–	–	–
*Scattering or scanning (63 reconstruction outcomes)*														
Jimenez 2020^†^	–	54	2/54	***4***	14/54	***26***	1/54	***2***	16/54	***30***	–	–	–	–
Jimenez 2019	55	53	–	–	12/51	***24***	12/51	***24***	5/51	***10***	–	–	1/2	***50***
**Subtotal**	**–**	**107**	**2/54**	***4***	**26/105**	***25***	**13/105**	***12***	**21/105**	***20***	**–**	**–**	**1/2**	***50***
*Scanning (77 reconstruction outcomes)*														
Mullikin 2020*	18	66	2/66	***3***	–	–	–	–	–	–	–	–	–	–
Gergelis 2021	–	62	–	–	–	–	–	–	–	–	–	–	–	–
Nichols 2020^†^	14	57	–	–	4/54	***7***	–	–	11/54	***20***	0/3	***0***	–	–
Smith 2019	19	51	16/51	***31***	1/51	***2***	–	–	8/51	***16***	–	–	–	–
Verma 2017	16	31	–	–	–	–	–	–	–	–	–	–	–	–
DeCesaris 2021^†^	5	29	–	–	–	–	–	–	2/29	***7***	1/29	***3***	–	–
Luo 2019	35	26	1/25	***4***	6/25	***24***	2/25	***8***	3/26	***12***	–	–	–	–
Naoum 2022^†‡^	–	17	–	–	8/17	***47***	–	–	9/17	***53***	–	–	–	–
Giap 2017	19	5	–	–	–	–	–	–	–	–	–	–	–	–
Anderson 2021^†§^	–	4	–	–	2/6	***33***	–	–	1/4	***25***	–	–	–	–
**Subtotal**	**–**	**348**	**19/142**	***13***	**21/153**	***14***	**2/25**	***8***	**34/181**	***19***	**1/32**	***3***	**–**	***–***
**Total** *(all PBT types)*	**–**	**459**	**22/200**	***11***	**47/258**	***18***	**15/130**	***12***	**55/286**	***19***	**1/32**	***3***	**1/2**	***50***

* Reported severe infections. For all other studies, infections were grade unspecified.

† Studies investigating proton beam therapy to the reconstructed breast only. Other studies reported outcomes in some patients with breast reconstructions and some without.

‡ Capsular contraction was defined as requiring capsulotomy. Capsular contraction was not defined in the other studies.

§ No. affected/assessed for capsular contraction were extracted from abstract where 6 patients with this outcome were reported upon.

Abbreviations: No.=number, recon=reconstructed breast. “-“=not specified.

## Data Availability

All data generated and analyzed during this study are included in this published article (and its supplementary information files).
