# Supplementary material for: Proton Beam Therapy for Early Breast Cancer: A Systematic Review and Meta-analysis of Clinical Outcomes
Source: Int J Radiat Oncol Biol Phys. Author manuscript; Available in PMC 2023 Nov 1. (PMC7615202; doi:10.1016/j.ijrobp.2023.02.023)
Supplement: Appendices [file EMS164673-supplement-Appendices.docx]

Proton Beam Therapy for Early Breast Cancer: A Systematic Review and Meta-analysis of Clinical Outcomes

Supplementary appendix

**Contents**

**Text A1:** Search Strategy 3

**Figure A1:** Process of study identification 4

**Table A1.** Studies analysing multi-institutional registry data on clinical outcomes after proton beam therapy for early breast cancer 6

**Table A2.** Definitions of grades of radiation dermatitis by grading system. 7

**Text A2:** References for Table 1 8

**Table A3.** Adverse outcomes excluded from main analysis 14

**Table A4.** Dermatitis after proton beam therapy for early breast cancer in studies published 2000-2022 according to clinical target and proton beam therapy type 16

**Table A5.** Adverse outcomes after proton beam therapy to the partial breast in studies published 2000-2022 according to proton beam therapy type 18

**Table A6.** Adverse outcomes after proton beam therapy to the whole breast or chest wall +/- regional lymph nodes in studies published 2000-2022 according to proton beam therapy type 22

**Figure A2.** Meta-analyses of the percentage of patients with moderate and mild dermatitis after scanning proton beam therapy to the whole breast or chest wall +/- regional lymph nodes. 28

**Table A7a.** Ongoing randomised studies of proton beam therapy for early breast cancer 29

**Table A7b.** Ongoing non-randomised studies of proton beam therapy for early breast cancer 30

# **Text A1: Search Strategy**

## **Published studies**

### Databases searched

Embase 1974 to 31^st^ August 2022

Ovid MEDLINE® 1946 to 1^st^ September 2022

### Search strategy

1 exp breast cancer/

2 (breast$ adj5 (neoplas$ or carcinom$ or cancer$ or tumor$ or tumour$)).af.

3 1 or 2

4 (advanced or metastatic or inoperable).ti.

5 locally advanced.af. or (neoadjuvant or adjuvant or early or operable).ti.

6 4 not 5

7 3 not 6

8 (proton$ and (therap$ or radiotherap$ or radiat$ or teletherap$)).af.

9 PBT.af.

10 8 or 9

11 7 and 10

12 human$/

13 nonhuman$/

14 13 not (12 and 13)

15 11 not 14

16 15

17 limit 16 to yr="2000 -Current"

## Abbreviations: exp=exploded term, $=unlimited truncation, adj5=terms next to each other, in any order, with up to 4 words in between, af=all fields, ti=title, yr=year

## **Ongoing studies**

### Databases searched

ClinicalTrials.gov, 30^th^ September 2022

World Health Organisation International Clinical Trials Registry Platform, 30^th^ October 2022

Cochrane Central Register of Controlled Trials, 30^th^ September 2022

### Search strategy

breast cancer AND proton

# **Figure A1: Process of study identification**

## **Published studies**

**338** duplicates

**3138** publications screened for eligibility

**3065** publications excluded:

- **3050** Not studies reporting clinical outcomes after adjuvant PBT for early breast cancer
- **2** Case reports
- **13** Clinical outcome data could not be extracted

**70** eligible publications of adjuvant PBT for early breast cancer reporting clinical outcomes grouped into **37** studies.

**32** individual studies included in analysis (1452 patients)

- **8** studies of PBT to the partial breast (358 patients)
- **19** studies of PBT to the whole breast or chest wall +/- regional lymph nodes (933 patients)
- **5** studies of PBT to the reconstructed breast +/- regional lymph nodes (161 patients)

**5** studies analysing registry data collated from multiple studies (1456 patients) excluded to prevent counting of individual patients more than once.

- **2** studies of PBT to the partial breast (378 patients)
- **3** studies of PBT to the whole breast or chest wall +/- regional lymph nodes (1078 patients)

(See Table A1)

**3476** publications identified through searching Ovid MEDLINE® and EMBASE

**73** eligible publications of adjuvant PBT for early breast cancer reporting clinical outcomes grouped into **37** studies.

## Abbreviation: PBT=proton beam therapy

## **Ongoing studi****es**

**175** studies identified through searching ClinicalTrials.gov, WHO Clinical Trials Registry Platform, and Cochrane Central Register of Controlled Trials September 2022

**27** duplicates

**148** studies screened for eligibility

**126** studies excluded:

- **120** studies did not investigate clinical outcomes after adjuvant PBT for early breast cancer
- **2** studies had been withdrawn since registration
- **4** studies were already included in the analysis of published studies.

**22** eligible ongoing studies of adjuvant PBT for early breast cancer collecting clinical outcomes summarised (estimated 9791 patients)

- **6** studies of PBT to the partial breast (765 patients)
- **11** studies of PBT to the whole breast or chest wall +/- regional lymph nodes (3583 patients)
- **5** studies of PBT to unspecified clinical target (5443 patients)

## Abbreviation: PBT=proton beam therapy

# **Table A1. Studies analysing multi-institutional registry data on clinical outcomes after proton beam therapy for early breast cancer**

| **Author and year of publication** | **Registry** | **Country** | **Year study started** | **Patients received PBT (N=1456)** |
| --- | --- | --- | --- | --- |
| **Partial breast (378 patients)** | | | | |
| *Scattering or scanning (335 patients)* | | | | |
| Fega 2017^1^ * | PCG | USA | 2011 | 335 |
| *Scanning (43 patients)* | | | | |
| Anderson 2019^2^ * | PCG | International | – | 43 |
| **Whole breast or chest wall +/- regional lymph nodes (1078 patients)** | | | | |
| *Scanning (82 patients)* | | | | |
| Thorpe 2020^3^ | PCG | USA | 2012 | 82 |
| *Type unspecified (996 patients)* | | | | |
| Chowdhary 2018^4^ | NCD | USA | 2004 | 871 |
| Niska 2019^5^ * | PCG | USA | 2011 | 125 |

**Footnotes**

These studies analysing registry data collated from multiple institutions were excluded from the review to prevent counting of individual patients more than once (see Figure A1(a)).

*Main study publication an abstract rather than a full text.

Abbreviations: N=total number, PBT=proton beam therapy, PCG=Proton Collaborative Group, NCD=National Cancer Database, USA=United States of America. “–“=not specified

**References for Table A1**

1.Fega R, Vargas CE, Hartsell WF, et al. Clinical outcomes of breast proton radiation therapy: A multi-institutional analysis of the proton collaborative group registry. *Int J Radiat Oncol Biol Phys*. 2017;99 (Suppl):E182. doi:10.1016/j.ijrobp.2017.06.527

2.Anderson J, Niska JR, Thorpe CS, et al. Proton Beam Accelerated Partial-Breast Irradiation: Prospective Multi-Institutional PCG Registry Analysis. *Int J Radiat Oncol Biol Phys*. 2019;105 (Suppl):E49. doi:10.1016/j.ijrobp.2019.06.2376

3.Thorpe CS, Niska JR, Anderson JD, et al. Acute toxicities after proton beam therapy following breast-conserving surgery for breast cancer: Multi-institutional prospective PCG registry analysis. *Breast J*. 2020;26:1760-64.

4.Chowdhary M, Barry P, Lee A, et al. Is proton therapy a "pro" for breast cancer? A comparison of proton vs. non-proton RT using the NCDB. *Radiother and Oncol*. 2019;133 (Suppl):S15. doi:10.1016/S0167-8140%2819%2930465-7

5.Niska JR, Thorpe CS, Anderson J, et al. Post-Mastectomy Radiotherapy using Proton Beam Therapy: Prospective Multi-Institutional PCG Registry Analysis. *Int J Radiat Oncol Biol Phys.* 2019;105 (Suppl):E6. doi:10.1016/j.ijrobp.2019.06.629

# **Table A2. Definitions of grades of radiation dermatitis by grading system.**

| **Grading system**  *(Definition)* | **Grade 1** | **Grade 2** | **Grade 3** |
| --- | --- | --- | --- |
| **CTCAE v5.0^1^ and v4.0^2^**  *(A finding of cutaneous inflammatory reaction occurring as a result of exposure to biologically effective levels of ionizing radiation.)* | Faint erythema or dry desquamation | Moderate to brisk erythema; patchy moist desquamation, mostly confined to skin folds and creases; moderate edema | Moist desquamation in areas other than skin folds and creases; bleeding induced by minor trauma or abrasion |
| **CTCAE v3.0^3^**  *(Dermatitis associated with radiation)* | Faint erythema or dry desquamation | Moderate to brisk erythema; patchy moist desquamation, mostly confined to skin folds and creases; moderate edema | Moist desquamation in areas other than skin folds and creases; bleeding induced by minor trauma or abrasion. |
| **CTC v2.0^4^**  *(Radiation dermatitis)* | Faint erythema or dry desquamation | Moderate to brisk erythema; patchy moist desquamation, mostly confined to skin folds and creases; moderate edema | Radiation dermatitis grade 3= confluent moist desquamation ≥1.5cm and not confined to skin folds; pitting oedema. |
| **RTOG/EORTC^5^**  *(Skin (acute))* | Follicular, faint or dull erythema/epilation/dry desquamation/decreased sweating. | Tender or bright erythema, patchy moist desquamation/moderate edema. | Confluent, moist desquamation other than skin folds, pitting edema. |

**Footnotes**

Abbreviations: CTCAE=Common Terminology Criteria for Adverse Events, CTC= Common Toxicity Criteria, EORTC= European Organisation for Research and Treatment of Cancer, RTOG=Radiation Therapy Oncology Group, v=version

**References for Table A2**

1. CTCAE version 5.0. NCI. <https://ctep.cancer.gov/protocoldevelopment/electronic_applications/docs/CTCAE_v5_Quick_Reference_5x7.pdf> Accessed September 20, 2022.
2. CTCAE version 4.03. NCI. [https://evs.nci.nih.gov/ftp1/CTCAE/CTCAE_4.03/CTCAE_4.03_2010-06-14_QuickReference_5x7.pdf Accessed Sept 2022](https://evs.nci.nih.gov/ftp1/CTCAE/CTCAE_4.03/CTCAE_4.03_2010-06-14_QuickReference_5x7.pdf%20Accessed%20Sept%202022) Accessed September 20, 2022.
3. CTCAE version 3.0. NCI. https://ctep.cancer.gov/protocoldevelopment/electronic_applications/docs/ctcaev3.pdf Accessed September 20, 2022.
4. CTC version 2.0. NCI. <https://ctep.cancer.gov/protocoldevelopment/electronic_applications/docs/ctcv20_4-30-992.pdf> Accessed September 20, 2022.
5. Cox JD, Stetz J, Pajak TF. Toxicity criteria of the Radiation Therapy Oncology Group (RTOG) and the European organization for research and treatment of cancer (EORTC). *Int J Radiat Oncol Biol Phys* 1995;31:1341-46. doi:10.1016/0360-3016(95)00

# **Text A2: References for Table 1**

1.Pasalic D, Strom EA, Allen PK, et al. Proton Accelerated Partial Breast Irradiation: Clinical Outcomes at a Planned Interim Analysis of a Prospective Phase 2 Trial. *Int J Radiat Oncol Biol Phys*. 2021;109:441-8. doi:10.1016/j.ijrobp.2020.09.009

2.Ovalle V, Strom EA, Shaitelman S, et al. Proton Partial Breast Irradiation: Detailed Description of Acute Clinico-Radiologic Effects. *Cancers (Basel)*. 2018;10:111 doi:10.3390/cancers10040111

3.Pasalic D, Strom EA, Allen PK, et al. Prospectively Assessed Outcomes for Proton Accelerated Partial Breast Irradiation. *Int J Radiat Oncol Biol Phys*. 2019;105 (Suppl):S193. doi:10.1016/j.ijrobp.2019.06.247

4.Bush DA, Slater JD, Garberoglio C, Do S, Lum S, Slater JM. Partial breast irradiation delivered with proton beam: results of a phase II trial. *Clin Breast Cancer*. 2011;11:241-5. doi:10.1016/j.clbc.2011.03.023

5.Bush DA, Slater JD, Garberoglio C, Do S, Lum S, Slater JM. Partial Breast Irradiation Delivered with Proton Beam: Results of a Phase II Trial. *Int J Radiat Oncol Biol Phys*. 2010;78 (Suppl): S157. doi:10.1016/j.ijrobp.2010.07.388

6.Bush DA, Do S, Lum S, et al. Partial breast radiation therapy with proton beam: 5-year results with cosmetic outcomes. *Int J Radiat Oncol Biol Phys*. 2014;90:501-5. doi:10.1016/j.ijrobp.2014.05.1308

7.Bush DA, Teichman SL, Do SY, Grove R, Davis C, Slater JD. Long-Term Quality of Life Outcomes of Proton Beam PBI Compared With Photon Whole Breast Radiation Therapy. *Int J Radiat Oncol Biol Phys*. 2017;99 (Suppl):E5. doi:10.1016/j.ijrobp.2017.06.604

8.Teichman SL, Do S, Lum S, et al. Improved long-term patient-reported health and well-being outcomes of early-stage breast cancer treated with partial breast proton therapy. *Cancer Med*. 2018;7:6064-76. doi:10.1002/cam4.1881

9.Kozak KR, Smith BL, Adams J, et al. Accelerated partial-breast irradiation using proton beams: initial clinical experience. *Int J Radiat Oncol Biol Phys*. 2006;66:691-8. doi: 10.1016/j.ijrobp.2006.06.041.

10.Galland-Girodet S, Pashtan I, MacDonald SM, et al. Long-term cosmetic outcomes and toxicities of proton beam therapy compared with photon-based 3-dimensional conformal accelerated partial-breast irradiation: a phase 1 trial. *Int J Radiat Oncol Biol Phys*. 2014;90:493-500. doi:10.1016/j.ijrobp.2014.04.008

11.Recht A, Ancukiewicz M, Alm El-Din MA, et al. Lung dose-volume parameters and the risk of pneumonitis for patients treated with accelerated partial-breast irradiation using three-dimensional conformal radiotherapy. *J Clin Oncol*. 2009;27:3887-93. doi:https://dx.doi.org/10.1200/JCO.2008.20.0121

12.Pashtan IM, Recht A, Ancukiewicz M, et al. External beam accelerated partial-breast irradiation using 32 gy in 8 twice-daily fractions: 5-year results of a prospective study. *Int J Radiat Oncol Biol Phys*. 2012;84:e271-7. doi:10.1016/j.ijrobp.2012.04.019

13.Galland S, Pashtan IM, MacDonald SM, et al. Long-term Cosmetic Outcomes and Toxicities of Proton Compared to Photon 3D-Conformal Accelerated Partial Breast Irradiation (3D-APBI). *Int J Radiat Oncol Biol Phys.* 2013;87 (Suppl):S195-6. doi:10.1016/j.ijrobp.2013.06.505

14.Mutter RW, Jethwa KR, Gonuguntla K, et al. 3 fraction pencil-beam scanning proton accelerated partial breast irradiation: early provider and patient reported outcomes of a novel regimen. *Radiat Oncol*. 2019;14:211. doi:10.1186/s13014-019-1417-7

15.Mutter RW, Jethwa KR, Gonuguntla K, et al. Abstracts from the NIH Office of Research on Women's Health 2019 Annual BIRCWH Meeting – Building Interdisciplinary Research Careers in Women's Health December 11, 2019. *Journal of Women's Health*. 2019;28:1577-96. doi:10.1089/jwh.2019.29030.abstracts

16.Choi JI, Prabhu K, Hartsell WF, et al. Outcomes and toxicities after proton partial breast radiotherapy for early stage, hormone receptor positive breast cancer: 3-Year results of a phase II multi-center trial. *Clin Transl Radiat Oncol*. Nov 2022;37:71-7. doi:10.1016/j.ctro.2022.08.013

17.Choi JI, Chang AL. Excellent acute toxicity outcomes with proton therapy for partialbreast irradiation in early stage breast cancer: Initial results of a multi-institutional phase II trial. *Cancer Res*. 2018;78 (Suppl): P2-11-09. doi:10.1158/15387445.SABCS17-P2-11-09

18.Choi IJ, Prabhu K, Hartsell WF, et al. Clinical Outcomes after Proton Partial-Breast Radiotherapy for Early-Stage, Hormone Receptor-Positive Breast Cancer: 3-Year Outcomes of a Phase II Trial. *Int J Radiat Oncol Biol Phys*. 2019;105 (Suppl):E19

doi:10.1016/j.ijrobp.2019.06.659

19.Giap F, Lepage R, Dong L, Giap HB. Intensity Modulated Proton Therapy for Accelerated Partial Breast Irradiation: A Single Institution Experience. *Int J Radiat Oncol Biol Phys*. 2017;99 (Suppl):E17. doi:10.1016/j.ijrobp.2017.06.630

20.Sckolnik SE, Simpson DR, Mascia AE, Lepage R, Giap H. Scanning beam intensity modulated proton therapy for accelerated partial-breast irradiation. *Int J Radiat Oncol Biol Phys*. 2015;93 (Suppl):E29.

21.Chang JH, Lee NK, Kim JY, et al. Phase II trial of proton beam accelerated partial breast irradiation in breast cancer. *Radiother Oncol*. 2013;108:209-14. doi:10.1016/j.radonc.2013.06.008

22.Shin K, Chang J, Moon S, et al. Clinical outcomes of proton beam therapy for accelerated partial breast irradiation. *Eur J Cancer*. 2012;48 (Suppl):S181. doi:10.1016/S0959-8049(12)70526-9

23.Sayan M, Jan I, Vergalasova I, et al. Incidence of clinical lymphedema in breast cancer patients treated with adjuvant proton-based radiotherapy. *Rep Pract Oncol Radiother*. 2022;27:474-8. doi:10.5603/RPOR.a2022.0053

24.Sayan M, Zhang Y, Liu B, Gupta A, Haffty B, Ohri N. Early toxicity and patient-reported cosmetic outcomes in patients treated with adjuvant proton-based radiotherapy after breast-conserving surgery. *Cancer Res*. 2020;80 (Suppl):P4-12-20 doi: 10.1158/1538-7445.SABCS19-P4-12-20

25.Liang X, Bradley JA, Zheng D, et al. Prognostic factors of radiation dermatitis following passive-scattering proton therapy for breast cancer. *Radiat Oncol*. 2018;13:72. doi:10.1186/s13014-018-1004-3

26.Bradley JA, Dagan R, Ho MW, et al. Initial Report of a Prospective Dosimetric and Clinical Feasibility Trial Demonstrates the Potential of Protons to Increase the Therapeutic Ratio in Breast Cancer Compared With Photons. *Int J Radiat Oncol Biol Phys*. 2016;95:411-21. doi:10.1016/j.ijrobp.2015.09.018

27.Bradley JA, Dagan R, Ho MW, et al. Pilot Study of Proton Therapy for Treatment of Regional Lymphatics in Breast Cancer Patients. *Int J Radiat Oncol Biol Phys*. 2013;87:S235. doi:10.1016/j.ijrobp.2013.06.609

28.Bradley JA, Dagan R, Ho MW, et al. Pilot study of proton therapy for treatment of regional lymphatics in patients with breast cancer. *J Clin Oncol*. 2013;31 (Suppl):100 doi:10.1200/jco.2013.31.26_suppl.100

29.Bradley JA, Dagan R, Ho MW, Rutenberg MS, Li Z, Mendenhall NP. Two-year outcomes of a prospective study of proton therapy for breast cancer regional nodal irradiation. *J Clin Oncol*. 2015;33 (Suppl):65 doi:10.1200/jco.2015.33.28_suppl.65

30.Bradley JA, Dagan R, Ho MW, et al. Two year outcomes of a prospective study of proton therapy for breast cancer regional nodal irradiation. *Int J Radiat Oncol Biol Phys*. 2015;93 (Suppl):E3. doi: 10.1016/j.ijrobp.2015.07.551

31.Bradley JA, Dagan R, Ho MW, Morris CG, Li Z, Mendenhall NP. Prospective study of proton radiotherapy for treatment of regional lymphatics in breast cancer. *Cancer Res*. 2015;75 (Suppl):P1-15-20 doi:10.1158/1538-7445.SABCS14-P1-15-20

32.Bradley JA, Dagan R, Liang X, et al. Five year outcomes of a prospective study of proton radiotherapy for breast cancer regional nodal irradiation. *Cancer Res*. 2019;79 (Suppl): P3-12-16 doi:10.1158/1538-7445.SABCS18-P3-12-16

33.Bradley JA, Liang X, Mailhot-Vega RB, et al. Three Year Outcomes of Proton Therapy for Regional Nodal Irradiation in Breast Cancer. *Int J Radiat Oncol Biol Phys*. 2019;105 (Suppl):E1. doi:10.1016/j.ijrobp.2019.06.617

34.Jimenez RB, Hickey S, DePauw N, et al. Phase II Study of Proton Beam Radiation Therapy for Patients With Breast Cancer Requiring Regional Nodal Irradiation. *J Clin Oncol:* 2019;37:2778-85. doi:10.1200/JCO.18.02366

35.MacDonald S, Specht M, Isakoff S, et al. Prospective Pilot Study of Proton Radiation Therapy for Invasive Carcinoma of the Breast Following Mastectomy in Patients With Unfavorable Anatomy -- First Reported Clinical Experience. *Int J Radiat Oncol Biol Phys*. 2012;84:S113-4. doi:10.1016/j.ijrobp.2012.07.196

36.MacDonald SM, Jimenez R, Paetzold P, et al. Proton radiotherapy for chest wall and regional lymphatic radiation; dose comparisons and treatment delivery. *Radiat Oncol*. 2013;8:71. doi:10.1186/1748-717X-8-71

37.MacDonald SM, Patel SA, Hickey S, et al. Proton therapy for breast cancer after mastectomy: early outcomes of a prospective clinical trial. *Int J Radiat Oncol Biol Phys*. 2013;86:484-90. doi:10.1016/j.ijrobp.2013.01.038

38.Naoum GE, Ho AY, Shui A, et al. Risk of Developing Breast Reconstruction Complications: A Machine-Learning Nomogram for Individualized Risk Estimation with and without Postmastectomy Radiation Therapy. *Plast Reconstr Surg*. 2022;149:1e-12e. doi:10.1097/PRS.0000000000008635

39.Mullikin TC, Aziz K, Afzal A, et al. Early Outcomes and Adverse Events following Post-Mastectomy Intensity Modulated Proton Therapy. *Int J Radiat Oncol Biol Phys*. 2020;108:e48. doi:10.1016/j.ijrobp.2020.07.1092

40.Zheng Y, Prabhu K, Larson GL, Vargas CE. Acute and late toxicity of uniform scanning proton therapy for breast cancer patients. *Int J Radiat Oncol Biol Phys*. 2017;99 (Suppl):E60. doi:https://dx.doi.org/10.1016/j.ijrobp.2017.06.734

41.DeCesaris C, Pollock A, Kowalski E, et al. Initial outcomes of adjuvant protonpencil beam scanning radiation for patients with breastcancer requiring comprehensive nodal irradiation within asingle institution. *Cancer Res*. 2021;81 (Suppl):PS15-12 doi:10.1158/1538-7445.SABCS20-PS15-12

42.Gergelis KR, Mullikin TC, Afzal A, et al. Post-Mastectomy Acute Skin Toxicity Assessment Between Particle and Photon Therapy. *Int J Radiat Oncol Biol Phys*. 2021;111 (Suppl):e222. doi:10.1016/j.ijrobp.2021.07.768

43.Verma V, Iftekaruddin Z, Badar N, et al. Proton beam radiotherapy as part of comprehensive regional nodal irradiation for locally advanced breast cancer. *Radiother Oncol*. 2017;123:294-8. doi:10.1016/j.radonc.2017.04.007.

44.Hartsell WF, Dorn PL, McGee LA, et al. Proton beam therapy for locally advanced breast cancer: Dosimetric results and acute toxicity. *Int J Radiat Oncol Biol Phys*. 2014;90 (Suppl):S273. doi:10.1016/j.ijrobp.2014.05.936

45.McGee LA, McGue MM, Dunn M, et al. (P022) Proton Therapy on an Incline Beam Line: Acute Toxicity Outcomes in Locally Advanced Breast Cancer Patients. *Proceedings of the 97th Annual Meeting of the American Radium Society*. 2015;29 (Suppl):205104

46.Iftekaruddin Z, McGee LA, Maliekel J, Dunn M, Hartsell WF. Acute toxicity outcomes in breast cancer patients treated with adjuvant proton therapy. *Int J Radiat Oncol Biol Phys*. 2015;93 (Suppl):E29-30. doi:10.1016/j.ijrobp.2015.07.618

47.Smith NL, Jethwa KR, Viehman JK, et al. Post-mastectomy intensity modulated proton therapy after immediate breast reconstruction: Initial report of reconstruction outcomes and predictors of complications. *Radiother Oncol*. 2019;140:76-83. doi:10.1016/j.radonc.2019.05.022

48.Mutter RW, Remmes NB, Kahila MM, et al. Initial clinical experience of postmastectomy intensity modulated proton therapy in patients with breast expanders with metallic ports. *Pract Radiat Oncol*. 2017;7:e243-52. doi:10.1016/j.prro.2016.12.002

49.Luo L, Cuaron J, Braunstein L, et al. Early outcomes of breast cancer patients treated with post-mastectomy uniform scanning proton therapy. *Radiother Oncol*. 2019;132:250-6. doi:10.1016/j.radonc.2018.10.002

50.Cuaron JJ, Hug E, Chon BH, Tsai H, Powell SN, Cahlon O. Outcomes and early toxicity in patients treated with proton therapy for breast cancer. *Int J Radiat Oncol Biol Phys*. 2014;90 (Suppl):S220. doi:10.1016/j.ijrobp.2014.05.798

51.Cuaron JJ, Chon B, Tsai H, et al. Early toxicity in patients treated with postoperative proton therapy for locally advanced breast cancer. *Int J Radiat Oncol Biol Phys*. 2015;92:284-91. doi:10.1016/j.ijrobp.2015.01.005

52.Luo L, Cuaron JJ, Braunstein LZ, et al. Outcome and late toxicity of breast cancer patients treated with post-mastectomy proton therapy. *Int J Radiat Oncol Biol Phys*. 2017;99 (Suppl):E33-4. doi:10.1016/j.ijrobp.2017.06.671

53.Pasztorova A, Kubes J, Andrlik M, et al. Breast cancer irradiation using proton pencil beam scanning. *Radiother Oncol*. 2018;127 (Suppl):S699-700. doi:10.1016/S0167-8140(18)31578-0

54.DeCesaris CM, Rice SR, Bentzen SM, Jatczak J, Mishra MV, Nichols EM. Quantification of Acute Skin Toxicities in Patients With Breast Cancer Undergoing Adjuvant Proton versus Photon Radiation Therapy: A Single Institutional Experience. *Int J Radiat Oncol Biol Phys*. 2019;104:1084-90. doi:10.1016/j.ijrobp.2019.04.015

55.Salari K, Quinn TJ, Ding X, Abbott V, Chen PY, Dilworth JT. Near-Surface Dose Correlates With Toxicity in Patients Receiving Pencil Beam Scanning Intensity Modulated Proton Beam Breast Irradiation. *Int J Radiat Oncol Biol Phys*. 2021;111 (Suppl):e225. 2021 doi:10.1016/j.ijrobp.2021.07.775

56.Cuaron JJ, Luo L, Braunstein LZ, et al. Cosmetic outcomes and toxicity in patients treated with proton therapy after breast conserving surgery. *Int J Radiat Oncol Biol Phys*. 2017;99 (Suppl):E11. doi: 10.1016/j.ijrobp.2017.06.617

57.Verhoeven K, Opbroek T, Vilches-Freixas G, et al. Prospectively registered acute toxicity in breast cancer patients undergoing adjuvant intensity modulated proton therapy. *Eur J Cancer*. 2020;138 (Suppl):S85. doi:10.1016/S0959-8049%2820%2930762-0

58.Fattahi S, Mullikin TC, Aziz KA, et al. Proton therapy for the treatment of inflammatory breast cancer. *Radiother Oncol*. 2022;171:77-83. doi:https://dx.doi.org/10.1016/j.radonc.2022.04.008

59.Fattahi S, Ahmed SK, Park SS, et al. Reirradiation of Locoregional Disease in Breast Cancer. *Int J Radiat Oncol Biol Phys*. 2020;108 (Suppl):e27-28. doi:10.1016/j.ijrobp.2020.07.1050

60.Garda AE, Hunzeker AE, Michel AK, et al. Intensity Modulated Proton Therapy for Bilateral Breast or Chest Wall and Comprehensive Nodal Irradiation for Synchronous Bilateral Breast Cancer: Initial Clinical Experience and Dosimetric Comparison. *Adv Radiat Oncol*. 2022;7:100901. doi:10.1016/j.adro.2022.100901

61.Loap P, Beddok A, Cao KI, et al. Clinical practice of breast cancer protontherapy: A single-centre experience from selection to treatment. *Cancer Radiother*. 2021;25:358-65. doi:10.1016/j.canrad.2021.01.005

62.Jimenez RB, Melick E, Naoum G, et al. Reconstructive outcomes among breast cancer patients receiving post-mastectomy radiation with protons versus conventional radiation. *Cancer Res*. 2020;80 (Suppl):P4-12-04 doi:10.1158/1538-7445.SABCS19-P4-12-04

63.Nichols EM, DeCesaris C, Pollock AE, Paulosky K, Mishra MV. Reconstructive Outcomes in Women Treated with Adjuvant Proton Therapy for Breast Cancer with Immediate Reconstruction at the Time of Mastectomy. *Int J Radiat Oncol Biol Phys*. 2020;108 (Suppl):e51. doi:10.1016/j.ijrobp.2020.07.1099

64.DeCesaris CM, Mossahebi S, Jatczak J, et al. Outcomes of and treatment planning considerations for a hybrid technique delivering proton pencil-beam scanning radiation to women with metal-containing tissue expanders undergoing post-mastectomy radiation. *Radiother Oncol*. 2021;164:289-98. doi:10.1016/j.radonc.2021.07.012

65.Naoum GE, Ioakeim MI, Shui AM, et al. Radiation Modality (Proton/Photon), Timing, and Complication Rates in Patients With Breast Cancer Receiving 2-Stages Expander/Implant Reconstruction. *Pract Radiat Oncol*. 2022 doi:10.1016/j.prro.2022.05.017

66.Naoum GE, Ioakeim-Ioannidou M, Shui AM, Salama L, Taghian AG. Analysis of Radiation Modality and Timing on Complication Rates in Breast Cancer Patients Receiving Two-Stage Expander/Implant Reconstruction. *Int J Radiat Oncol Biol Phys*. 2021;111 (Suppl):S107-8. doi:10.1016/j.ijrobp.2021.07.248

67.Anderson JD, Hammond JB, Kosiorek HE, et al. Unplanned implant removal in locally advanced breast cancer. *Breast J*. 2021;27(5):466-71. doi:10.1111/tbj.14224

68.Anderson J, Hammond JB, Thorpe CS, et al. Capsular Contracture after Radiation Therapy Following Mastectomy and Implant-Based Reconstruction. *Int J Radiat Oncol Biol Phys*. 2019;105 (Suppl):E50. doi:10.1016/j.ijrobp.2019.06.23

| **Table A3. Adverse outcomes excluded from main analysis** | | | | | | | | | | | | |
| --- | --- | --- | --- | --- | --- | --- | --- | --- | --- | --- | --- | --- |
| **Author and year of publication** | **Adverse outcome** | **Grading system** | **Reason for exclusion** | **Time assessed post PBT (months)** | **Severe** | | **Moderate** | | **Mild** | | **Unspecified** | |
|  |  |  |  |  | **Affected/ Assessed** | ***%*** | **Affected/ Assessed** | ***%*** | **Affected/ Assessed** | ***%*** | **Affected/ Assessed** | ***%*** |
| **Partial breast** | | | | | | | | | | | | |
| *Scattering* | | | | | | | | | | | | |
| Pasalic 2021 | Pruritis | CTCAE v4.0 | a | 2 | 0/100 | ***0*** | 1/100 | ***1*** | 29/100 | ***29*** | *–* | **–** |
| Kozak 2006 | Moist desquamation | mild/moderate/  severe | a | 2 | 2/19 | ***11*** | 2/19 | ***11*** | 0/19 | ***0*** | *–* | **–** |
| *Scanning* | | | | | | | | | | | | |
| Choi 2022 | Pruritus | CTCAE v4.0 | a | 35 | 0/38 | ***0*** | 0/38 | ***0*** | 1/38 | ***3*** | *–* | **–** |
|  | Vascular ischaemia | CTCAE v4.0 | e | 1 | 1/38 | ***3*** | 0/38 | ***0*** | 0/38 | ***0*** | *–* | **–** |
|  | Hot flashes | CTCAE v4.0 | e | – | 0/38 | ***0*** | 3/38 | ***8*** | 10/38 | ***26*** | *–* | **–** |
|  | Dyspnoea | CTCAE v4.0 | e | – | 0/38 | ***0*** | 1/38 | ***3*** | 0/38 | ***0*** | *–* | **–** |
|  | Cough | CTCAE v4.0 | e | – | 0/38 | ***0*** | 0/38 | ***0*** | 3/38 | ***8*** | *–* | **–** |
|  | Back pain | CTCAE v4.0 | e | – | 0/38 | ***0*** | 0/38 | ***0*** | 2/38 | ***5*** | *–* | **–** |
|  | Diarrhoea | CTCAE v4.0 | e | – | 0/38 | ***0*** | 0/38 | ***0*** | 1/38 | ***3*** | *–* | **–** |
|  | Arthralgia | CTCAE v4.0 | e | – | 0/38 | ***0*** | 0/38 | ***0*** | 2/38 | ***5*** | *–* | **–** |
| Giap 2017 | Dry skin | – | a | – | – | **–** | – | **–** | 3/25 | ***12*** | *–* | **–** |
| Mutter 2019 | Seroma | CTCAE v4.0 | e | 0 | 0/76 | ***0*** | 0/76 | ***0*** | 25/76 | ***33*** | *–* | **–** |
| **Whole breast or Chest wall +/- regional lymph nodes** | | | | | | | | | | | | |
| *Scattering* | | | | | | | | | | | | |
| Bradley 2016 | Nausea | CTCAE v4.0 | e | – | 0/18 | ***0*** | 1/18 | ***6*** | *–* | **–** | – | **–** |
| *Scattering or scanning* | | | | | | | | | | | | |
| Jimenez 2019 | Seroma | CTCAE v4.0 | e | – | 1/69 | ***1*** | 0/69 | ***0*** | 0/69 | ***0*** | *–* | **–** |
| *Scanning* | | | | | | | | | | | | |
| Fattahi 2022 | Wound dehiscence | CTCAE v5.0 | e | – | – | **–** | – | **–** | – | **–** | 1/19 | ***5*** |
| Garda 2022 | Limb oedema | CTCAE v4.0 | c | 32 | 0/11 | ***0*** | 0/11 | ***0*** | 2/11 | ***18*** | *–* | **–** |
| Pasztorova 2018 | Acute toxicity | RTOG | d | – | 1/42 | ***2*** | 18/42 | ***43*** | 23/42 | ***55*** | *–* | **–** |
|  | Late toxicity | RTOG | d | – | 3/42 | ***7*** | 7/42 | ***17*** | 12/42 | ***29*** | *–* | **–** |
| Smith 2019 | Haematoma | – | e | – | – | **–** | – | **–** | – | **–** | 1/51 | ***2*** |
|  | Seroma |  | e | – | – | **–** | – | **–** | – | **–** | 4/51 | ***8*** |
|  | Flap necrosis |  | e | – | – | **–** | – | **–** | – | **–** | 2/51 | ***4*** |
| Zheng 2017 | Hot flashes | – | e | >6 | 0/100 | ***0*** | 2/100 | ***2*** | 1/100 | ***1*** | *–* | **–** |
|  | Arthralgia |  | e | >6 | 0/100 | ***0*** | 0/100 | ***0*** | 2/100 | ***2*** | *–* | **–** |
|  | Nipple deformation |  | b | >6 | 0/100 | ***0*** | 0/100 | ***0*** | 2/100 | ***2*** | – | ***–*** |
| *Continued on next page* | | | | | | | | | | | | |
| **Table A3. Adverse outcomes excluded from main analysis *(continued)*** | | | | | | | | | | | | |
| **Footnotes** | | | | | | | | | | | | |
| a = May not be distinct from other included counts of dermatitis. | | | | | | | | | | | | |
| b = May or may not be distinct from other skin- or soft tissue- related adverse outcomes.  c = May or may not be distinct from included lymphoedema | | | | | | | | | | | | |
| d = Toxicity could not be categorised further. | | | | | | | | | | | | |
| e = Study authors categorised as unlikely related to radiotherapy. | | | | | | | | | | | | |
| Abbreviations: CTCAE=Common Terminology Criteria for Adverse Events, PBT=proton beam therapy, RTOG=Radiation Therapy Oncology Group, v=version, “–“=not specified | | | | | | | | | | | | |

# **Table A4. Dermatitis after proton beam therapy for early breast cancer in studies published 2000-2022 according to clinical target and proton beam therapy type**

| **Author and year of publication** | **PBT total dose (Gy)/ No. fractions** | **Dermatitis** | | | | | | | | |
| --- | --- | --- | --- | --- | --- | --- | --- | --- | --- | --- |
|  |  | **Grading system** | **Time assessed post PBT (months)** | **Total no. assessed for dermatitis** | **Severe** | | **Moderate** | | **Mild** | |
|  |  |  |  |  | **No. affected** | ***%*** | **No. affected** | ***%*** | **No. affected** | ***%*** |
| **Partial breast** | | | | | | | | | | |
| *Scattering* | | | | | | | | | | |
| Kozak 2006 | 32/8* | mild/moderate/severe | 2 | 19 | 7 | ***37*** | 3 | ***16*** | 4 | ***21*** |
| Galland-Girodet 2014 | 32/8* | RTOG/EORTC | 60 | 19 | – | **–** | 8 | ***42*** | – | **–** |
| Pasalic 2021 | 34/10 | CTCAE v4.0 | 1.5 | 100 | 0 | ***0*** | 11 | ***11*** | 56 | ***56*** |
| Bush 2011 | 40/10 | CTC v2.0 | <3 | 50 | 0 | ***0*** | 4 | ***8*** | 26 | ***52*** |
| **Subtotal (partial breast, scattering)** | | | | **188** | 7 | ***4*** | 26 | ***14*** | 86 | ***51*** |
| *Scanning* | | | | | | | | | | |
| Giap 2017 | 40/10 | – | – | 25 | – | **–** | 3 | ***12*** | – | **–** |
| Choi 2022 | 40/10 | CTCAE v4.0 | 35 | 38 | 0 | ***0*** | 1 | ***3*** | 27 | ***71*** |
| Mutter 2019 | 22/3 | CTCAE v4.0 | 0 | 76 | 0 | ***0*** | 0 | ***0*** | 52 | ***68*** |
| **Subtotal (partial breast, scanning)** | | | | **139** | 0 | ***0*** | 4 | ***3*** | 79 | ***69*** |
| *Type unspecified* | | | | | | | | | | |
| Chang 2013 | 30/5 | mild/moderate/severe | 2 | **30** | 1 | ***3*** | 1 | ***3*** | 6 | ***20*** |
| **Total (partial breast)** |  |  |  | **357** | 8 | ***3*** | 31 | ***9*** | 171 | ***55*** |
| *Continued on next page.* | | | | | | | | | | |
| **Table A4. Dermatitis after proton beam therapy for early breast cancer in studies published 2000-2022 according to clinical target and proton beam therapy type *(continued)*** | | | | | | | | | | |
| **Author and year of publication** | **PBT total dose (Gy)/ No. fractions** | **Dermatitis** | | | | | | | | |
|  |  | **Grading system** | **Time assessed post PBT (months)** | **Total no. assessed for dermatitis** | **Severe** | | **Moderate** | | **Mild** | |
|  |  |  |  |  | **No. affected** | ***%*** | **No. affected** | ***%*** | **No. affected** | ***%*** |
| **Whole breast or Chest wall +/- regional lymph nodes** | | | | | | | | | | |
| *Scattering* | | | | | | | | | | |
| Bradley 2016 | 50/28 | CTCAE v4.0 | <1 | 18 | 1 | ***6*** | 17 | ***94*** | ***–*** | **–** |
| Sayan 2022 | 40-50/– | CTCAE v3.0 | – | 28 | 4 | ***14*** | 19 | ***68*** | ***–*** | **–** |
| Liang 2018 | 50/25-28 | CTCAE v4.0 | – | 23 | 10 | ***43*** | 13 | ***57*** | ***–*** | **–** |
| **Subtotal (whole breast or chest wall +/- regional lymph nodes, scattering)** | | | | **69** | 15 | ***22*** | 49 | ***71*** | **–** | ***–*** |
| *Scattering or scanning* | | | | | | | | | | |
| Jimenez 2019 | 45-50/25-28 | CTCAE v4.0 | – | **69** | 2 | ***3*** | 57 | ***83*** | 10 | ***14*** |
| *Scanning* | | | | | | | | | | |
| Gergelis 2021 | 50/25 | CTCAE v4.0 | – | 96 | 3 | ***3*** | 42 | ***44*** | – | – |
| Mullikin 2020 | 50/25 | CTCAE v5.0 | – | 126 | 8 | ***6*** | 58 | ***46*** | 60 | ***48*** |
| Smith 2019 | 50/25 | CTCAE v4.0 | – | 51 | 2 | ***4*** | 17 | ***33*** | 32 | ***63*** |
| Luo 2019 | 50/– | CTCAE v4.0 | <3 | 42 | 0 | ***0*** | 31 | ***74*** | 11 | ***26*** |
| Verma 2017 | 50/25-28 | CTCAE v4.0 | – | 93 | 5 | ***5*** | 67 | ***72*** | 21 | ***23*** |
| Garda 2022 | 50/25 | CTCAE v4.0 | 0 | 11 | 1 | ***9*** | 5 | ***45*** | 5 | ***45*** |
| Loap 2021 | 50/25 | – | – | 1 | – | **–** | 1 | ***100*** | *–* | **–** |
| DeCesaris 2021 | 50/25-28 | – | – | 100 | 10^†^ | ***10*** | – | **–** | – | **–** |
| Verhoeven 2020 | 40/15 | CTCAE v4.0 | 1 | 20 | 2 | ***10*** | 16 | ***80*** | 2 | ***10*** |
| Fattahi 2022 | 50/25 | CTCAE v5.0 | – | 19 | 2 | ***11*** | 17 | ***89*** | – | **–** |
| DeCesaris 2019 | 45-50/25-28 | CTCAE v4.0 | – | 39 | 2 | ***5*** | 25 | ***64*** | 12 | ***31*** |
| Zheng 2017 | 45-50/25-28 | – | <6 | 100 | 6 | ***6*** | 52 | ***52*** | 31 | ***31*** |
| Cuaron 2017 | 49/– | CTCAE v4.0 | – | 26 | 0 | ***0*** | 12 | ***46*** | – | **–** |
| **Subtotal (whole breast or chest wall +/- regional lymph nodes, scanning)** | | | | **724** | 41 | ***6*** | 343 | ***55*** | 174 | ***36*** |
| **Total (whole breast or chest wall +/- regional lymph nodes)** | | |  | **862** | 58 | ***7*** | 449 | ***52*** | 184 | ***21*** |
| **Total (all studies)** |  |  |  | **1219** | 66 | ***6*** | 480 | ***43*** | 355 | ***41*** |

**Footnotes**

Individual studies reporting dermatitis of a specified grade after PBT for early breast cancer. Summary percentages for each type of PBT are calculated using the total number of patients assessed for each grade of dermatitis. The total number of patients vary as not all studies assessed all grades of dermatitis.

Salari 2021 reported 5/16 patients with unspecified grade of dermatitis. These patients were excluded from this analysis.

*Two fractions delivered daily. In all other studies where duration of treatment was reported, one fraction was delivered daily.

†One of the 10 severe adverse events was graded 4 for the reason of skin necrosis. All other severe adverse events were grade 3.

Abbreviations: CTCAE=Common Terminology Criteria for Adverse Events, EORTC=European Organisation for Research and Treatment of Cancer, Gy=gray, No.=number, PBT=proton beam therapy, RTOG=Radiation Therapy Oncology Group, v=version, “–“=not specified

| **Table A5. Adverse outcomes after proton beam therapy to the partial breast in studies published 2000-2022 according to proton beam therapy type** | | | | | | | | | | | | | | | | | | | | | | | | | | | | | | | | | | | | |
| --- | --- | --- | --- | --- | --- | --- | --- | --- | --- | --- | --- | --- | --- | --- | --- | --- | --- | --- | --- | --- | --- | --- | --- | --- | --- | --- | --- | --- | --- | --- | --- | --- | --- | --- | --- | --- |
| **Author and year of publication** | **PBT dose(Gy)/ No. fractions** | | | **Grading system** | | | **Time assessed post PBT (months)** | | | **Total no. assessed** | | | **Severe** | | | | | | **Moderate** | | | | | | **Mild** | | | | | | **Unspecified** | | | | | |
|  |  |  |  |  |  |  |  |  |  |  |  |  | **No. affected** | | | ***%*** | | | **No. affected** | | | ***%*** | | | **No. affected** | | | ***%*** | | | **No. affected** | | | ***%*** | | |
| **Atrophy** | | | | | | | | | | | | | | | | | | | | | | | | | | | | | | | | | | | | |
| *Scattering* | | | | | | | | | | | | | | | | | | | | | | | | | | | | | | | | | | | | |
| Galland-Girodet 2014 | 32/8* | | | RTOG/EORTC | | | 84 | | | 19 | | | – | | | **–** | | | 10 | | | ***53*** | | | *–* | | | **–** | | | – | | | **–** | | |
| **Total** |  | | |  | | |  | | | **19** | | | – | | | ***–*** | | | 10 | | | ***53*** | | | – | | | ***–*** | | | – | | | ***–*** | | |
| **Breast oedema** | | | | | | | | | | | | | | | | | | | | | | | | | | | | | | | | | | | | |
| *Scattering* | | | | | | | | | | | | | | | | | | | | | | | | | | | | | | | | | | | | |
| Pasalic 2021 | 34/10* | | | CTCAE v4.0 | | | 2 | | | 100 | | | 0 | | | ***0*** | | | 0 | | | ***0*** | | | 10 | | | ***10*** | | | *–* | | | **–** | | |
| Kozak 2006 | 32/8* | | | mild/moderate/severe | | | 1 | | | 19 | | | 0 | | | ***0*** | | | 1 | | | ***5*** | | | 5 | | | ***26*** | | | *–* | | | **–** | | |
| *Scanning* | | | | | | | | | | | | | | | | | | | | | | | | | | | | | | | | | | | | |
| Mutter 2019 | 22/3 | | | CTCAE v4.0 | | | 0 | | | 76 | | | 0 | | | ***0*** | | | 0 | | | ***0*** | | | 17 | | | ***22*** | | | *–* | | | **–** | | |
| **Total** |  | | |  | | |  | | | **195** | | | 0 | | | ***0*** | | | 1 | | | ***1*** | | | 32 | | | ***16*** | | | – | | | ***–*** | | |
| **Cardiac** | | | | | | | | | | | | | | | | | | | | | | | | | | | | | | | | | | | | |
| *Scattering* | | | | | | | | | | | | | | | | | | | | | | | | | | | | | | | | | | | | |
| Bush 2011 | 40/10 | | | – | | | – | | | 50 | | | – | | | **–** | | | – | | | **–** | | | – | | | **–** | | | 0 | | | ***0*** | | |
| **Total** |  | | |  | | |  | | | **50** | | | – | | | ***–*** | | | – | | | ***–*** | | | – | | | ***–*** | | | 0 | | | ***0*** | | |
| **Fat necrosis** | | | | | | | | | | | | | | | | | | | | | | | | | | | | | | | | | | | | |
| *Scattering* | | | | | | | | | | | | | | | | | | | | | | | | | | | | | | | | | | | | |
| Pasalic 2021 | 34/10* | | | CTCAE v.40 | | | 36 | | | 100 | | | 0 | | | ***0*** | | | 0 | | | ***0*** | | | 0 | | | ***0*** | | |  | | | **–** | | |
| Galland-Girodet 2014 | 32/8* | | | – | | | – | | | 19 | | | – | | | **–** | | | – | | | **–** | | | – | | | **–** | | | 2 | | | ***11*** | | |
| Bush 2011 | 40/10 | | | – | | | – | | | 50 | | | 0 | | | ***0*** | | | 0 | | | ***0*** | | | 0 | | | ***0*** | | | 0 | | | ***0*** | | |
| Kozak 2006 | 32/8* | | | – | | | 12 | | | 13 | | | 0 | | | ***0*** | | | 1 | | | ***8*** | | | 0 | | | ***0*** | | | *–* | | | **–** | | |
| **Total** |  | | |  | | |  | | | **163** | | | 0 | | | ***0*** | | | 1 | | | ***1*** | | | **0** | | | ***0*** | | | 2 | | | ***3*** | | |
| *Continued on next page.* | | | | | | | | | | | | | | | | | | | | | | | | | | | | | | | | | | | | |
| **Table A5. Adverse outcomes after proton beam therapy to the partial breast in studies published 2000-2022 according to proton beam therapy type *(continued)*** | | | | | | | | | | | | | | | | | | | | | | | | | | | | | | | | | | | | |
| **Author and year of publication** | **PBT dose(Gy)/ No. fractions** | | | **Grading system** | | | **Time assessed post PBT (months)** | | | **Total no. assessed** | | | **Severe** | | | | | | **Moderate** | | | | | | **Mild** | | | | | | **Unspecified** | | | | | |
|  |  |  |  |  |  |  |  |  |  |  |  |  | **No. affected** | | | ***%*** | | | **No. affected** | | | ***%*** | | | **No. affected** | | | ***%*** | | | **No. affected** | | | ***%*** | | |
|  | | | | | | | | | | | | | | | | | | | | | | | | | | | | | | | | | | | | |
| **Fatigue** | | | | | | | | | | | | | | | | | | | | | | | | | | | | | | | | | | | | |
| *Scattering* | | | | | | | | | | | | | | | | | | | | | | | | | | | | | | | | | | | | |
| Pasalic 2021 | 34/10* | | | CTCAE v4.0 | | | 12 | | | 100 | | | 0 | | | ***0*** | | | 4 | | | ***4*** | | | 10 | | | ***10*** | | | *–* | | | **–** | | |
| *Scanning* | | | | | | | | | | | | | | | | | | | | | | | | | | | | | | | | | | | | |
| Choi 2022 | 40/10 | | | CTCAE v4.0 | | | 35 | | | 38 | | | 0 | | | ***0*** | | | 1 | | | ***3*** | | | 8 | | | ***21*** | | | *–* | | | **–** | | |
| Giap 2017 | 40/10 | | | – | | | – | | | 5 | | | – | | | **–** | | | – | | | **–** | | | 5 | | | ***100*** | | | *–* | | | **–** | | |
| **Total** |  | | |  | | |  | | | **143** | | | 0 | | | ***0*** | | | 5 | | | ***4*** | | | 23 | | | ***16*** | | | – | | | ***–*** | | |
| **Fibrosis‡** | | | | | | | | | | | | | | | | | | | | | | | | | | | | | | | | | | | | |
| *Scattering* | | | | | | | | | | | | | | | | | | | | | | | | | | | | | | | | | | | | |
| Galland-Girodet 2014 | 32/8* | | | RTOG/EORTC | | | – | | | 19 | | | – | | | **–** | | | – | | | **–** | | | – | | | **–** | | | 10 | | | ***53*** | | |
| Kozak 2006 | 32/8* | | | mild/moderate/severe | | | 1 | | | 19 | | | 0 | | | ***0*** | | | 1 | | | ***5*** | | | 3 | | | ***16*** | | | *–* | | | **–** | | |
| *Scanning* | | | | | | | | | | | | | | | | | | | | | | | | | | | | | | | | | | | | |
| Mutter 2019 | 22/3 | | | CTCAE v4.0 | | | 0 | | | 76 | | | 0 | | | ***0*** | | | 0 | | | ***0*** | | | 1 | | | ***1*** | | | *–* | | | **–** | | |
| *Type unspecified* | | | | | | | | | | | | | | | | | | | | | | | | | | | | | | | | | | | | |
| Chang 2013 | 30/5 | | | mild/moderate/severe | | | 12 | | | 30 | | | 0 | | | ***0*** | | | 2 | | | ***7*** | | | 7 | | | ***23*** | | | – | | | ***–*** | | |
| **Total** |  | | |  | | |  | | | **144** | | | 0 | | | ***0*** | | | 3 | | | ***2*** | | | 11 | | | ***9*** | | | 10 | | | ***53*** | | |
| **Hyperpigmentation** | | | | | | | | | | | | | | | | | | | | | | | | | | | | | | | | | | | | |
| *Scattering* | | | | | | | | | | | | | | | | | | | | | | | | | | | | | | | | | | | | |
| Pasalic 2021 | 34/10* | | | CTCAE v4.0 | | | 1.5 | | | 100 | | | 0 | | | ***0*** | | | 2 | | | ***2*** | | | 44 | | | ***44*** | | | *–* | | | **–** | | |
| *Scanning* | | | | | | | | | | | | | | | | | | | | | | | | | | | | | | | | | | | | |
| Mutter 2019 | 22/3 | | | CTCAE v4.0 | | | 3 | | | 72 | | | 0 | | | ***0*** | | | 0 | | | ***0*** | | | 13 | | | ***18*** | | | *–* | | | **–** | | |
| *Type unspecified* | | | | | | | | | | | | | | | | | | | | | | | | | | | | | | | | | | | | |
| Chang 2013 | 30/5 | | | mild/moderate/severe | | | 2 | | | 30 | | | 0 | | | ***0*** | | | 9 | | | ***30*** | | | 21 | | | ***70*** | | | *–* | | | **–** | | |
| **Total** |  | | |  | | |  | | | **202** | | | 0 | | | ***0*** | | | 11 | | | ***5*** | | | 78 | | | ***39*** | | |  | | |  | | |
| *Continued on next page.* | | | | | | | | | | | | | | | | | | | | | | | | | | | | | | | | | | | | |
| **Table A5. Adverse outcomes after proton beam therapy to the partial breast in studies published 2000-2022 according to proton beam therapy type *(continued)*** | | | | | | | | | | | | | | | | | | | | | | | | | | | | | | | | | | | | |
| **Author and year of publication** | | **PBT dose(Gy)/ No. fractions** | | | **Grading system** | | | **Time assessed post PBT (months)** | | | **Total no. assessed** | | | **Severe** | | | | | | **Moderate** | | | | | | **Mild** | | | | | | **Unspecified** | | | | |
|  |  |  |  |  |  |  |  |  |  |  |  |  |  | **No. affected** | | | ***%*** | | | **No. affected** | | | ***%*** | | | **No. affected** | | | ***%*** | | | **No. affected** | | | ***%*** | |
| **Infection** | | | | | | | | | | | | | | | | | | | | | | | | | | | | | | | | | | | | |
| *Scattering* | | | | | | | | | | | | | | | | | | | | | | | | | | | | | | | | | | | | |
| Pasalic 2021 | 34/10* | | | CTCAE v4.0 | | | 1 | | | 100 | | | 0 | | | ***0*** | | | 1 | | | ***1*** | | | 0 | | | ***0*** | | | – | | | **–** | | |
| Bush 2011 | 40/10 | | | – | | | – | | | 50 | | | – | | | **–** | | | – | | | **–** | | | – | | | **–** | | | 0 | | | ***0*** | | |
| *Scanning* | | | | | | | | | | | | | | | | | | | | | | | | | | | | | | | | | | | | |
| Mutter 2019 | 22/3 | | | CTCAE v4.0 | | | 0 | | | 76 | | | 0 | | | ***0*** | | | 0 | | | ***0*** | | | 0 | | | ***0*** | | | – | | | **–** | | |
| **Total** |  | | |  | | |  | | | **226** | | | 0 | | | ***0*** | | | 1 | | | ***1*** | | | 0 | | | ***0*** | | | 0 | | | ***0*** | | |
| **Lymphoedema** | | | | | | | | | | | | | | | | | | | | | | | | | | | | | | | | | | | | |
| *Scanning* | | | | | | | | | | | | | | | | | | | | | | | | | | | | | | | | | | | | |
| Choi 2022 | 40/10 | | | CTCAE v4.0 | | | 35 | | | 38 | | | 0 | | | ***0*** | | | 1 | | | ***3*** | | | 2 | | | ***5*** | | | – | | | **–** | | |
| **Total** |  | | |  | | |  | | | **38** | | | 0 | | | ***0*** | | | 1 | | | ***3*** | | | 2 | | | ***5*** | | | – | | | **–** | | |
| **Pain§** | | | | | | | | | | | | | | | | | | | | | | | | | | | | | | | | | | | | |
| *Scattering* | | | | | | | | | | | | | | | | | | | | | | | | | | | | | | | | | | | | |
| Pasalic 2021 | 34/10* | | | CTCAE v4.0 | | | 1.5 | | | 100 | | | 0 | | | ***0*** | | | 1 | | | ***1*** | | | 27 | | | ***27*** | | | – | | | **–** | | |
| Galland-Girodet 2014 | 32/8* | | | RTOG/EORTC | | | 36 | | | 19 | | | – | | | **–** | | | – | | | **–** | | | – | | | **–** | | | 4 | | | ***21*** | | |
| Kozak 2006 | 32/8* | | | mild/moderate/severe | | | 1 | | | 19 | | | 0 | | | ***0*** | | | 2 | | | ***11*** | | | 7 | | | ***37*** | | | – | | | **–** | | |
| *Scanning* | | | | | | | | | | | | | | | | | | | | | | | | | | | | | | | | | | | | |
| Mutter 2019 | 22/3 | | | CTCAE v4.0 | | | 0 | | | 76 | | | 0 | | | ***0*** | | | 0 | | | ***0*** | | | 4 | | | ***5*** | | | – | | | **–** | | |
| Choi 2022 | 40/10 | | | CTCAE v4.0 | | | 35 | | | 38 | | | 0 | | | ***0*** | | | 0 | | | ***0*** | | | 5 | | | ***13*** | | | – | | | **–** | | |
| *Type unspecified* | | | | | | | | | | | | | | | | | | | | | | | | | | | | | | | | | | | | |
| Chang 2013 | 30/5 | | | mild/moderate/severe | | | 2 | | | 30 | | | 0 | | | ***0*** | | | 0 | | | ***0*** | | | 11 | | | ***37*** | | | – | | | **–** | | |
| **Total** |  | | |  | | |  | | | **282** | | | 0 | | | ***0*** | | | 3 | | | ***1*** | | | 54 | | | ***21*** | | | 4 | | | ***21*** | | |
| **Pneumonitis ¦** | | | | | | | | | | | | | | | | | | | | | | | | | | | | | | | | | | | | |
| *Scattering* | | | | | | | | | | | | | | | | | | | | | | | | | | | | | | | | | | | | |
| Galland-Girodet 2014 | 32/8* | | | CTCAE v3.0 | | | – | | | 20 | | | 0 | | | ***0*** | | | 0 | | | ***0*** | | | 0 | | | ***0*** | | | – | | | **–** | | |
| Bush 2011 | 40/10 | | | – | | | – | | | 50 | | | – | | | **–** | | | – | | | **–** | | | – | | | **–** | | | 0 | | | ***0*** | | |
| *Scanning* | | | | | | | | | | | | | | | | | | | | | | | | | | | | | | | | | | | | |
| Mutter 2019 | 22/3 | | | CTCAE v4.0 | | | 0 | | | 76 | | | 0 | | | ***0*** | | | 0 | | | ***0*** | | | 0 | | | ***0*** | | | – | | | **–** | | |
| **Total** |  | | |  | | |  | | | **146** | | | 0 | | | ***0*** | | | 0 | | | ***0*** | | | 0 | | | ***0*** | | | 0 | | | ***0*** | | |
| *Continued on next page.* | | | | | | | | | | | | | | | | | | | | | | | | | | | | | | | | | | | | |
|  | | | | | | | | | | | | | | | | | | | | | | | | | | | | | | | | | | | | |
| **Table A5. Adverse outcomes after proton beam therapy to the partial breast in studies published 2000-2022 according to proton beam therapy type *(continued)*** | | | | | | | | | | | | | | | | | | | | | | | | | | | | | | | | | | | | |
| **Author and year of publication** | | | **PBT dose(Gy)/ No. fractions** | | | **Grading system** | | | **Time assessed post PBT (months)** | | | **Total no. assessed** | | | **Severe** | | | | | | **Moderate** | | | | | | **Mild** | | | | | | **Unspecified** | | | |
|  |  |  |  |  |  |  |  |  |  |  |  |  |  |  | **No. affected** | | | ***%*** | | | **No. affected** | | | ***%*** | | | **No. affected** | | | ***%*** | | | **No. affected** | | | ***%*** |
| **Rib fractures** | | | | | | | | | | | | | | | | | | | | | | | | | | | | | | | | | | | | |
| *Scattering* | | | | | | | | | | | | | | | | | | | | | | | | | | | | | | | | | | | | |
| Galland-Girodet 2014 | 32/8* | | | – | | | 60 | | | 19 | | | – | | | **–** | | | – | | | **–** | | | – | | | **–** | | | 1 | | | ***5*** | | |
| Bush 2011 | 40/10* | | | – | | | – | | | 50 | | | – | | | **–** | | | – | | | **–** | | | – | | | **–** | | | 0 | | | ***0*** | | |
| Kozak 2006 | 32/8* | | | – | | | 6 | | | 20 | | | – | | | **–** | | | – | | | **–** | | | – | | | **–** | | | 1 | | | ***5*** | | |
| *Scanning* | | | | | | | | | | | | | | | | | | | | | | | | | | | | | | | | | | | | |
| Mutter 2019 | 22/3 | | | – | | | 12 | | | 76 | | | – | | | **–** | | | – | | | **–** | | | – | | | **–** | | | 0 | | | ***0*** | | |
| *Type unspecified* | | | | | | | | | | | | | | | | | | | | | | | | | | | | | | | | | | | | |
| Chang 2013 | 30/5 | | | Symptoms† | | | 24 | | | 30 | | | – | | | **–** | | | – | | | **–** | | | – | | | **–** | | | 2 | | | ***7*** | | |
| **Total** |  | | |  | | |  | | | **195** | | | – | | | **–** | | | – | | | **–** | | | – | | | **–** | | | **4** | | | ***2*** | | |
| **Telangiectasia** | | | | | | | | | | | | | | | | | | | | | | | | | | | | | | | | | | | | |
| *Scattering* | | | | | | | | | | | | | | | | | | | | | | | | | | | | | | | | | | | | |
| Pasalic 2021 | 34/10* | | | CTCAE v4.0 | | | 18 | | | 100 | | | – | | | **–** | | | – | | | **–** | | | 17 | | | ***17*** | | | – | | | – | | |
| Galland-Girodet 2014 | 32/8* | | | RTOG/EORTC | | | 84 | | | 19 | | | – | | | **–** | | | – | | | **–** | | | – | | | **–** | | | 7 | | | ***37*** | | |
| Bush 2011 | 40/10 | | | CTC v2.0 | | | – | | | 50 | | | – | | | **–** | | | – | | | **–** | | | 3 | | | ***6*** | | | – | | | **–** | | |
| Kozak 2006 | 32/8* | | | – | | | 24 | | | 20 | | | – | | | **–** | | | – | | | **–** | | | – | | | **–** | | | 3 | | | ***15*** | | |
| *Scanning* | | | | | | | | | | | | | | | | | | | | | | | | | | | | | | | | | | | | |
| Mutter 2019 | 22/3 | | | CTCAE v4.0 | | | 3 | | | 72 | | | 0 | | | ***0*** | | | 0 | | | ***0*** | | | 1 | | | ***1*** | | | – | | | **–** | | |
| Choi 2022 | 40/10 | | | CTCAE v4.0 | | | 35 | | | 38 | | | 0 | | | ***0*** | | | 0 | | | ***0*** | | | 1 | | | ***3*** | | | – | | | **–** | | |
| *Type unspecified* | | | | | | | | | | | | | | | | | | | | | | | | | | | | | | | | | | | | |
| Chang 2013 | 30/5 | | | – | | | – | | | – | | | – | | | **–** | | | – | | | **–** | | | – | | | **–** | | | – | | | **–** | | |
| **Total** |  | | |  | | |  | | | **299** | | | 0 | | | ***0*** | | | 0 | | | ***0*** | | | 22 | | | ***8*** | | | 10 | | | ***26*** | | |

**Footnotes**

Denominators for each study vary by adverse outcome as not all studies assessed all patients at all timepoints for all adverse outcomes.

*Two fractions delivered daily. In all other studies where duration of treatment was reported, one PBT fraction was delivered daily.

†Symptomatic with traumatic change on bone scanning without dislocation.

‡Fibrosis includes reports of induration.

§Includes reports of pain from skin, breast and unspecified pain.

¦Includes reports of cough and dyspnoea.

Abbreviations: CTCAE=Common Terminology Criteria for Adverse Events, CTC=Common Toxicity Criteria, Gy=Gray, No.=number, PBT=proton beam therapy, RTOG/EORTC=Radiation Therapy Oncology Group and the European organization for research and treatment of cancer, v=version. Blank or “–“=not specified

| **Table A6. Adverse outcomes after proton beam therapy to the whole breast or chest wall +/- regional lymph nodes in studies published 2000-2022 according to proton beam therapy type** | | | | | | | | | | | | | | | | | | | | | | | | | | | | | | | | | | | | | | | | | | | | | | | | | | | | | | | | | |
| --- | --- | --- | --- | --- | --- | --- | --- | --- | --- | --- | --- | --- | --- | --- | --- | --- | --- | --- | --- | --- | --- | --- | --- | --- | --- | --- | --- | --- | --- | --- | --- | --- | --- | --- | --- | --- | --- | --- | --- | --- | --- | --- | --- | --- | --- | --- | --- | --- | --- | --- | --- | --- | --- | --- | --- | --- | --- |
| **Author and year of publication** | **PBT dose(Gy)/ No. fractions** | | | | **Grading system** | | | | | **Time assessed post PBT (months)** | | | | | **Total no. assessed** | | | | | **Severe** | | | | | | | | | | **Moderate** | | | | | | | | | | **Mild** | | | | | | | | | | **Unspecified** | | | | | | | |
|  |  |  |  |  |  |  |  |  |  |  |  |  |  |  |  |  |  |  |  | **No. affected** | | | | | ***%*** | | | | | **No. affected** | | | | | ***%*** | | | | | **No. affected** | | | | | ***%*** | | | | | **No. affected** | | | | ***%*** | | | |
| **Atrophy** | | | | | | | | | | | | | | | | | | | | | | | | | | | | | | | | | | | | | | | | | | | | | | | | | | | | | | | | | |
| *Scattering* | | | | | | | | | | | | | | | | | | | | | | | | | | | | | | | | | | | | | | | | | | | | | | | | | | | | | | | | | |
| Bradley 2016 | 50/28 | | | | CTCAE v4.0 | | | | | – | | | | | 18 | | | | | 0 | | | | | ***0*** | | | | | 1 | | | | | ***6*** | | | | | – | | | | | **–** | | | | | – | | | | **–** | | | |
| *Scattering or scanning proton beam therapy* | | | | | | | | | | | | | | | | | | | | | | | | | | | | | | | | | | | | | | | | | | | | | | | | | | | | | | | | | |
| Jimenez 2019 | 45-50/25-28 | | | | CTCAE v4.0 | | | | | – | | | | | 69 | | | | | – | | | | | **–** | | | | | – | | | | | **–** | | | | | 1 | | | | | ***1*** | | | | | – | | | | **–** | | | |
| **Total** |  | | | |  | | | | |  | | | | | **87** | | | | | 0 | | | | | ***0*** | | | | | 1 | | | | | ***6*** | | | | | 1 | | | | | ***1*** | | | | | – | | | | **–** | | | |
| **Brachial plexopathy** | | | | | | | | | | | | | | | | | | | | | | | | | | | | | | | | | | | | | | | | | | | | | | | | | | | | | | | | | |
| *Scanning* | | | | | | | | | | | | | | | | | | | | | | | | | | | | | | | | | | | | | | | | | | | | | | | | | | | | | | | | | |
| Garda 2022 | 50/25 | | | | CTCAE v4.0 | | | | | 32 | | | | | 11 | | | | | 0 | | | | | ***0*** | | | | | 0 | | | | | ***0*** | | | | | 1 | | | | | ***9*** | | | | | – | | | | **–** | | | |
| Smith 2019 | 50/25 | | | | CTCAE v4.0 | | | | | 0 | | | | | 51 | | | | | 0 | | | | | ***0*** | | | | | 0 | | | | | ***0*** | | | | | 1 | | | | | ***2*** | | | | | – | | | | **–** | | | |
| Luo 2019 | 50/– | | | | CTCAE v4.0 | | | | | >3 | | | | | 42 | | | | | 0 | | | | | ***0*** | | | | | 0 | | | | | ***0*** | | | | | 0 | | | | | ***0*** | | | | | – | | | | **–** | | | |
| Zheng 2017 | 45-50/25-28 | | | | – | | | | | >6 | | | | | 100 | | | | | 0 | | | | | ***0*** | | | | | 0 | | | | | ***0*** | | | | | 1 | | | | | ***1*** | | | | | – | | | | **–** | | | |
| **Total** |  | | | |  | | | | |  | | | | | **204** | | | | | 0 | | | | | ***0*** | | | | | 0 | | | | | ***0*** | | | | | 3 | | | | | ***1*** | | | | | – | | | | **–** | | | |
| **Cardiac** | | | | | | | | | | | | | | | | | | | | | | | | | | | | | | | | | | | | | | | | | | | | | | | | | | | | | | | | | |
| *Scattering* | | | | | | | | | | | | | | | | | | | | | | | | | | | | | | | | | | | | | | | | | | | | | | | | | | | | | | | | | |
| Bradley 2016 | 50/28 | | | | CTCAE v4.0 | | | | | – | | | | | 18 | | | | | 0 | | | | | ***0*** | | | | | – | | | | | **–** | | | | | – | | | | | **–** | | | | | 2 | | | | ***11*** | | | |
| *Scanning* | | | | | | | | | | | | | | | | | | | | | | | | | | | | | | | | | | | | | | | | | | | | | | | | | | | | | | | | | |
| Loap 2021 | 50/25 | | | | – | | | | | – | | | | | 1 | | | | | 0 | | | | | ***0*** | | | | | 0 | | | | | ***0*** | | | | | 0 | | | | | ***0*** | | | | | – | | | | **–** | | | |
| DeCesaris 2021 | 50/25-28 | | | | – | | | | | – | | | | | 100 | | | | | 0 | | | | | ***0*** | | | | | 0 | | | | | ***0*** | | | | | 0 | | | | | ***0*** | | | | | – | | | | **–** | | | |
| Smith 2019 | 50/25 | | | | CTCAE v4.0 | | | | | – | | | | | 51 | | | | | 0 | | | | | ***0*** | | | | | 0 | | | | | ***0*** | | | | | 0 | | | | | ***0*** | | | | | – | | | | **–** | | | |
| Luo 2019 | 50/– | | | | CTCAE v4.0 | | | | | – | | | | | 42 | | | | | 0 | | | | | ***0*** | | | | | 0 | | | | | ***0*** | | | | | 0 | | | | | ***0*** | | | | | – | | | | **–** | | | |
| Cuaron 2017 | 49/– | | | | CTCAE v4.0 | | | | | – | | | | | 26 | | | | | 0 | | | | | ***0*** | | | | | 0 | | | | | ***0*** | | | | | 0 | | | | | ***0*** | | | | | – | | | | **–** | | | |
| **Total** |  | | | |  | | | | |  | | | | | **238** | | | | | 0 | | | | | ***0*** | | | | | 0 | | | | | ***0*** | | | | | 0 | | | | | ***0*** | | | | | 2 | | | | ***11*** | | | |
| **Decreased shoulder movement** | | | | | | | | | | | | | | | | | | | | | | | | | | | | | | | | | | | | | | | | | | | | | | | | | | | | | | | | | |
| *Scanning* | | | | | | | | | | | | | | | | | | | | | | | | | | | | | | | | | | | | | | | | | | | | | | | | | | | | | | | | | |
| Garda 2022 | 50/25 | | | | CTCAE v4.0 | | | | | 3 | | | | | 11 | | | | | 0 | | | | | ***0*** | | | | | 0 | | | | | ***0*** | | | | | 4 | | | | | ***36*** | | | | | – | | | | **–** | | | |
| Fattahi 2022 | 50/25 | | | | CTCAE v5.0 | | | | | – | | | | | 19 | | | | | – | | | | | **–** | | | | | 1 | | | | | ***5*** | | | | | 2 | | | | | ***11*** | | | | | – | | | | **–** | | | |
| **Total** |  | | | |  | | | | |  | | | | | **30** | | | | | 0 | | | | | ***0*** | | | | | 1 | | | | | ***3*** | | | | | 6 | | | | | ***20*** | | | | | – | | | | **–** | | | |
| *Continued on next page.* | | | | | | | | | | | | | | | | | | | | | | | | | | | | | | | | | | | | | | | | | | | | | | | | | | | | | | | | | |
| **Table A6. Adverse outcomes after proton beam therapy to the whole breast or chest wall +/- regional lymph nodes in studies published 2000-2022 according to proton beam therapy type *(continued)*** | | | | | | | | | | | | | | | | | | | | | | | | | | | | | | | | | | | | | | | | | | | | | | | | | | | | | | | | | |
| **Author and year of publication** | **PBT dose(Gy)/ No. fractions** | | | | **Grading system** | | | | | **Time assessed post PBT (months)** | | | | | **Total no. assessed** | | | | | **Severe** | | | | | | | | | | **Moderate** | | | | | | | | | | **Mild** | | | | | | | | | | **Unspecified** | | | | | | | |
|  |  |  |  |  |  |  |  |  |  |  |  |  |  |  |  |  |  |  |  | **No. affected** | | | | | ***%*** | | | | | **No. affected** | | | | | ***%*** | | | | | **No. affected** | | | | | ***%*** | | | | | **No. affected** | | | | ***%*** | | | |
| **Fatigue** | | | | | | | | | | | | | | | | | | | | | | | | | | | | | | | | | | | | | | | | | | | | | | | | | | | | | | | | | |
| *Scattering* | | | | | | | | | | | | | | | | | | | | | | | | | | | | | | | | | | | | | | | | | | | | | | | | | | | | | | | | | |
| Bradley 2016 | 50/28 | | | | CTCAE v4.0 | | | | | <3 | | | | | 18 | | | | | 0 | | | | | ***0*** | | | | | 6 | | | | | ***33*** | | | | | *–* | | | | | **–** | | | | | – | | | | **–** | | | |
| *Scattering or scanning* | | | | | | | | | | | | | | | | | | | | | | | | | | | | | | | | | | | | | | | | | | | | | | | | | | | | | | | | | |
| Jimenez 2019 | 45-50/25-28 | | | | CTCAE v4.0 | | | | | – | | | | | 69 | | | | | 0 | | | | | ***0*** | | | | | 24 | | | | | ***35*** | | | | | 38 | | | | | ***55*** | | | | | – | | | | **–** | | | |
| *Scanning* | | | | | | | | | | | | | | | | | | | | | | | | | | | | | | | | | | | | | | | | | | | | | | | | | | | | | | | | | |
| Garda 2022 | 50/25 | | | | CTCAE v4.0 | | | | | 0 | | | | | 11 | | | | | 0 | | | | | ***0*** | | | | | 1 | | | | | ***9*** | | | | | 7 | | | | | ***64*** | | | | | – | | | | **–** | | | |
| Luo 2019 | 50/– | | | | CTCAE v4.0 | | | | | <3 | | | | | 42 | | | | | 0 | | | | | ***0*** | | | | | 1 | | | | | ***2*** | | | | | 12 | | | | | ***29*** | | | | | – | | | | **–** | | | |
| Verma 2017 | 50/25-28 | | | | CTCAE v4.0 | | | | | – | | | | | 91 | | | | | 0 | | | | | ***0*** | | | | | 5 | | | | | ***5*** | | | | | 42 | | | | | ***46*** | | | | | – | | | | **–** | | | |
| **Total** |  | | | |  | | | | |  | | | | | **231** | | | | | 0 | | | | | ***0*** | | | | | 37 | | | | | ***16*** | | | | | 99 | | | | | ***46*** | | | | | – | | | | **–** | | | |
| **Fibrosis§** | | | | | | | | | | | | | | | | | | | | | | | | | | | | | | | | | | | | | | | | | | | | | | | | | | | | | | | | | |
| *Scanning* | | | | | | | | | | | | | | | | | | | | | | | | | | | | | | | | | | | | | | | | | | | | | | | | | | | | | | | | | |
| Garda 2022 | 50/25 | | | | CTCAE v4.0 | | | | | 32 | | | | | 11 | | | | | 0 | | | | | ***0*** | | | | | 0 | | | | | ***0*** | | | | | 4 | | | | | ***36*** | | | | | – | | | | **–** | | | |
| Fattahi 2022 | 50/25 | | | | CTCAE v5.0 | | | | | – | | | | | 19 | | | | | – | | | | | **–** | | | | | – | | | | | **–** | | | | | 2 | | | | | ***11*** | | | | | – | | | | **–** | | | |
| Cuaron 2017 | 49/– | | | | CTCAE v4.0 | | | | | – | | | | | 26 | | | | | – | | | | | **–** | | | | | 1 | | | | | ***4*** | | | | | 3 | | | | | ***12*** | | | | | – | | | | **–** | | | |
| **Total** |  | | | |  | | | | |  | | | | | **56** | | | | | 0 | | | | | ***0*** | | | | | 1 | | | | | ***3*** | | | | | 9 | | | | | ***16*** | | | | | – | | | | **–** | | | |
| **Hyperpigmentation** | | | | | | | | | | | | | | | | | | | | | | | | | | | | | | | | | | | | | | | | | | | | | | | | | | | | | | | | | |
| *Scattering or scanning* | | | | | | | | | | | | | | | | | | | | | | | | | | | | | | | | | | | | | | | | | | | | | | | | | | | | | | | | | |
| Jimenez 2019 | 45-50/25-28 | | | | CTCAE v4.0 | | | | | – | | | | | 69 | | | | | – | | | | | **–** | | | | | – | | | | | **–** | | | | | 36 | | | | | ***52*** | | | | | – | | | | **–** | | | |
| *Scanning* | | | | | | | | | | | | | | | | | | | | | | | | | | | | | | | | | | | | | | | | | | | | | | | | | | | | | | | | | |
| Garda 2022 | 50/25 | | | | CTCAE v4.0 | | | | | 3 | | | | | 11 | | | | | 0 | | | | | ***0*** | | | | | 0 | | | | | ***0*** | | | | | 8 | | | | | ***73*** | | | | | – | | | | **–** | | | |
| Fattahi 2022 | 50/25 | | | | CTCAE v5.1 | | | | | – | | | | | 19 | | | | | – | | | | | **–** | | | | | – | | | | | **–** | | | | | – | | | | | **–** | | | | | 8 | | | | ***42*** | | | |
| Mullikin 2020 | 50/25 | | | | CTCAE v5.0 | | | | | – | | | | | 126 | | | | | 0 | | | | | ***0*** | | | | | 0 | | | | | ***0*** | | | | | 55 | | | | | ***44*** | | | | | – | | | | **–** | | | |
| Luo 2019 | 50/– | | | | CTCAE v4.0 | | | | | >3 | | | | | 42 | | | | | – | | | | | **–** | | | | | 0 | | | | | ***0*** | | | | | 17 | | | | | ***40*** | | | | | – | | | | **–** | | | |
| DeCesaris 2019 | 45-50/25-28 | | | | CTCAE v4.0 | | | | | – | | | | | 39 | | | | | 0 | | | | | ***0*** | | | | | 3 | | | | | ***8*** | | | | | 14 | | | | | ***36*** | | | | | – | | | | **–** | | | |
| Cuaron 2017 | 49/– | | | | CTCAE v4.0 | | | | | – | | | | | 26 | | | | | – | | | | | **–** | | | | | – | | | | | **–** | | | | | 12 | | | | | ***46*** | | | | | – | | | | **–** | | | |
| **Total** |  | | | |  | | | | |  | | | | | **332** | | | | | 0 | | | | | ***0*** | | | | | 3 | | | | | ***1*** | | | | | 142 | | | | | ***45*** | | | | | 8 | | | | ***42*** | | | |
| *Continued on next page.* | | | | | | | | | | | | | | | | | | | | | | | | | | | | | | | | | | | | | | | | | | | | | | | | | | | | | | | | | |
|  | | | | | | | | | | | | | | | | | | | | | | | | | | | | | | | | | | | | | | | | | | | | | | | | | | | | | | | | | |
| **Table A6. Adverse outcomes after proton beam therapy to the whole breast or chest wall +/- regional lymph nodes in studies published 2000-2022 according to proton beam therapy type *(continued)*** | | | | | | | | | | | | | | | | | | | | | | | | | | | | | | | | | | | | | | | | | | | | | | | | | | | | | | | | | |
| **Author and year of publication** | | **PBT dose(Gy)/ No. fractions** | | | | **Grading system** | | | | | **Time assessed post PBT (months)** | | | | | **Total no. assessed** | | | | | **Severe** | | | | | | | | | | **Moderate** | | | | | | | | | | **Mild** | | | | | | | | | | **Unspecified** | | | | | | |
|  |  |  |  |  |  |  |  |  |  |  |  |  |  |  |  |  |  |  |  |  | **No. affected** | | | | | ***%*** | | | | | **No. affected** | | | | | ***%*** | | | | | **No. affected** | | | | | ***%*** | | | | | **No. affected** | | | | ***%*** | | |
| **Infection** | | | | | | | | | | | | | | | | | | | | | | | | | | | | | | | | | | | | | | | | | | | | | | | | | | | | | | | | | |
| *Scattering* | | | | | | | | | | | | | | | | | | | | | | | | | | | | | | | | | | | | | | | | | | | | | | | | | | | | | | | | | |
| Bradley 2016 | 50/28 | | | | CTCAE v4.0 | | | | | – | | | | | 18 | | | | | 3 | | | | | ***17*** | | | | | 3 | | | | | ***17*** | | | | | *–* | | | | | **–** | | | | | – | | | | **–** | | | |
| *Scattering or scanning* | | | | | | | | | | | | | | | | | | | | | | | | | | | | | | | | | | | | | | | | | | | | | | | | | | | | | | | | | |
| Jimenez 2019 | 45-50/25-28 | | | | CTCAE v4.0 | | | | | 4 | | | | | 69 | | | | | 1 | | | | | ***1*** | | | | | 0 | | | | | ***0*** | | | | | 0 | | | | | ***0*** | | | | | *–* | | | | **–** | | | |
| *Scanning* | | | | | | | | | | | | | | | | | | | | | | | | | | | | | | | | | | | | | | | | | | | | | | | | | | | | | | | | | |
| Garda 2022 | 50/25 | | | | CTCAE v4.0 | | | | | 32 | | | | | 11 | | | | | 0 | | | | | ***0*** | | | | | 0 | | | | | ***0*** | | | | | 0 | | | | | ***0*** | | | | | *–* | | | | **–** | | | |
| Mullikin 2020 | 50/25 | | | | CTCAE v5.0 | | | | | – | | | | | 126 | | | | | 1 | | | | | ***1*** | | | | | 0 | | | | | ***0*** | | | | | 0 | | | | | ***0*** | | | | | *–* | | | | **–** | | | |
| Fattahi 2022 | 50/25 | | | | CTCAE v5.0 | | | | | – | | | | | 19 | | | | | – | | | | | **–** | | | | | 2 | | | | | ***11*** | | | | | *–* | | | | | **–** | | | | | – | | | | **–** | | | |
| Verma 2017 | 50/25-28 | | | | CTCAE v4.0 | | | | | – | | | | | 91 | | | | | – | | | | | **–** | | | | | – | | | | | **–** | | | | | – | | | | | **–** | | | | | 7 | | | | ***8*** | | | |
| **Total** |  | | | |  | | | | |  | | | | | **334** | | | | | 5 | | | | | ***2*** | | | | | 5 | | | | | ***2*** | | | | | 0 | | | | | ***0*** | | | | | 7 | | | | ***8*** | | | |
| **Lymphoedema** | | | | | | | | | | | | | | | | | | | | | | | | | | | | | | | | | | | | | | | | | | | | | | | | | | | | | | | | | |
| *Scattering* | | | | | | | | | | | | | | | | | | | | | | | | | | | | | | | | | | | | | | | | | | | | | | | | | | | | | | | | | |
| Sayan 2022 | 40-50/– | | | | CTCAE v3.0 | | | | | – | | | | | 28 | | | | | – | | | | | **–** | | | | | – | | | | | **–** | | | | | – | | | | | **–** | | | | | 5 | | | | ***18*** | | | |
| Bradley 2016 | 50/28 | | | | CTCAE v4.0 | | | | | – | | | | | 18 | | | | | 0 | | | | | ***0*** | | | | | 1 | | | | | ***6*** | | | | | *–* | | | | | **–** | | | | | – | | | | **–** | | | |
| *Scattering or scanning* | | | | | | | | | | | | | | | | | | | | | | | | | | | | | | | | | | | | | | | | | | | | | | | | | | | | | | | | | |
| Jimenez 2019 | 45-50/25-28 | | | | CTCAE v4.0 | | | | | – | | | | | 69 | | | | | 0 | | | | | ***0*** | | | | | 0 | | | | | ***0*** | | | | | 1 | | | | | ***1*** | | | | | *–* | | | | **–** | | | |
| *Scanning* | | | | | | | | | | | | | | | | | | | | | | | | | | | | | | | | | | | | | | | | | | | | | | | | | | | | | | | | | |
| Garda 2022 | 50/25 | | | | CTCAE v4.0 | | | | | 0 | | | | | 11 | | | | | 0 | | | | | ***0*** | | | | | 0 | | | | | ***0*** | | | | | 2 | | | | | ***18*** | | | | | *–* | | | | **–** | | | |
| Fattahi 2022 | 50/25 | | | | CTCAE v5.0 | | | | | – | | | | | 19 | | | | | – | | | | | **–** | | | | | 3 | | | | | ***16*** | | | | | 5 | | | | | ***26*** | | | | | *–* | | | | **–** | | | |
| Luo 2019 | 50/– | | | | CTCAE v4.0 | | | | | >3 | | | | | 42 | | | | | 0 | | | | | ***0*** | | | | | 0 | | | | | ***0*** | | | | | 12 | | | | | ***29*** | | | | | *–* | | | | **–** | | | |
| Zheng 2017 | 45-50/25-28 | | | | – | | | | | >6 | | | | | 100 | | | | | 0 | | | | | ***0*** | | | | | 0 | | | | | ***0*** | | | | | 2 | | | | | ***2*** | | | | | *–* | | | | **–** | | | |
| Verma 2017 | 50/25-28 | | | | CTCAE v4.0 | | | | | – | | | | | 91 | | | | | – | | | | | **–** | | | | | – | | | | | **–** | | | | | – | | | | | **–** | | | | | 3 | | | | ***3*** | | | |
| **Total** |  | | | |  | | | | |  | | | | | **378** | | | | | 0 | | | | | ***0*** | | | | | 4 | | | | | ***2*** | | | | | 22 | | | | | ***9*** | | | | | 8 | | | | ***7*** | | | |
| *Continued on next page.* | | | | | | | | | | | | | | | | | | | | | | | | | | | | | | | | | | | | | | | | | | | | | | | | | | | | | | | | | |
| **Table A6. Adverse outcomes after proton beam therapy to the whole breast or chest wall +/- regional lymph nodes in studies published 2000-2022 according to proton beam therapy type *(continued)*** | | | | | | | | | | | | | | | | | | | | | | | | | | | | | | | | | | | | | | | | | | | | | | | | | | | | | | | | | |
| **Author and year of publication** | | | **PBT dose(Gy)/ No. fractions** | | | | **Grading system** | | | | | **Time assessed post PBT (months)** | | | | | **Total no. assessed** | | | | | **Severe** | | | | | | | | | | **Moderate** | | | | | | | | | | **Mild** | | | | | | | | | | **Unspecified** | | | | | |
|  |  |  |  |  |  |  |  |  |  |  |  |  |  |  |  |  |  |  |  |  |  | **No. affected** | | | | | ***%*** | | | | | **No. affected** | | | | | ***%*** | | | | | **No. affected** | | | | | ***%*** | | | | | **No. affected** | | | | ***%*** | |
| **Oesophagitis†** | | | | | | | | | | | | | | | | | | | | | | | | | | | | | | | | | | | | | | | | | | | | | | | | | | | | | | | | | |
| *Scattering* | | | | | | | | | | | | | | | | | | | | | | | | | | | | | | | | | | | | | | | | | | | | | | | | | | | | | | | | | |
| Sayan 2022 | 40-50/– | | | | CTCAE v3.0 | | | | | – | | | | | 28 | | | | | – | | | | | **–** | | | | | 1 | | | | | ***4*** | | | | | – | | | | | **–** | | | | | – | | | | **–** | | | |
| Bradley 2016 | 50/28 | | | | CTCAE v4.0 | | | | | – | | | | | 18 | | | | | 0 | | | | | ***0*** | | | | | 5 | | | | | ***28*** | | | | | – | | | | | **–** | | | | | – | | | | **–** | | | |
| *Scattering or scanning* | | | | | | | | | | | | | | | | | | | | | | | | | | | | | | | | | | | | | | | | | | | | | | | | | | | | | | | | | |
| Jimenez 2019 | 45-50/25-28 | | | | CTCAE v4.0 | | | | | – | | | | | 69 | | | | | 0 | | | | | ***0*** | | | | | 5 | | | | | ***7*** | | | | | 19 | | | | | ***28*** | | | | | – | | | | **–** | | | |
| *Scanning* | | | | | | | | | | | | | | | | | | | | | | | | | | | | | | | | | | | | | | | | | | | | | | | | | | | | | | | | | |
| Garda 2022 | 50/25 | | | | CTCAE v4.0 | | | | | 0 | | | | | 11 | | | | | 0 | | | | | ***0*** | | | | | 0 | | | | | ***0*** | | | | | 1 | | | | | ***9*** | | | | | – | | | | **–** | | | |
| Verhoeven 2020 | 40/15 | | | | CTCAE v4.0 | | | | | 1 | | | | | 15 | | | | | 0 | | | | | ***0*** | | | | | 4 | | | | | ***27*** | | | | | 4 | | | | | ***27*** | | | | | – | | | | **–** | | | |
| Mullikin 2020 | 50/25 | | | | CTCAE v5.0 | | | | | – | | | | | 126 | | | | | 0 | | | | | ***0*** | | | | | 0 | | | | | ***0*** | | | | | 9 | | | | | ***7*** | | | | | – | | | | **–** | | | |
| Fattahi 2022 | 50/25 | | | | CTCAE v5.0 | | | | | – | | | | | 19 | | | | |  | | | | |  | | | | | 1 | | | | | ***5*** | | | | | 2 | | | | | ***11*** | | | | | – | | | | **–** | | | |
| Smith 2019 | 50/25 | | | | CTCAE v4.0 | | | | | – | | | | | 51 | | | | | 0 | | | | | ***0*** | | | | | 1 | | | | | ***2*** | | | | | 3 | | | | | ***6*** | | | | | – | | | | **–** | | | |
| Luo 2019 | 50/– | | | | CTCAE v4.0 | | | | | <3 | | | | | 42 | | | | | 0 | | | | | ***0*** | | | | | 7 | | | | | ***17*** | | | | | 15 | | | | | ***36*** | | | | | – | | | | **–** | | | |
| Zheng 2017 | 45-50/25-28 | | | | – | | | | | <6 | | | | | 100 | | | | | – | | | | | **–** | | | | | – | | | | | **–** | | | | | – | | | | | **–** | | | | | 2 | | | | ***2*** | | | |
| Verma 2017 | 50/25-28 | | | | CTCAE v4.0 | | | | | – | | | | | 91 | | | | | 0 | | | | | ***0*** | | | | | 30 | | | | | ***33*** | | | | | 28 | | | | | ***31*** | | | | | – | | | | **–** | | | |
| **Total** |  | | | |  | | | | |  | | | | | **570** | | | | | 0 | | | | | ***0*** | | | | | 54 | | | | | ***11*** | | | | | 81 | | | | | ***19*** | | | | | 2 | | | | ***2*** | | | |
| **Pain*** | | | | | | | | | | | | | | | | | | | | | | | | | | | | | | | | | | | | | | | | | | | | | | | | | | | | | | | | | |
| *Scattering* | | | | | | | | | | | | | | | | | | | | | | | | | | | | | | | | | | | | | | | | | | | | | | | | | | | | | | | | | |
| Sayan 2022 | 40-50/– | | | | CTCAE v3.0 | | | | | – | | | | | 28 | | | | | – | | | | | **–** | | | | | 9 | | | | | ***32*** | | | | | – | | | | | **–** | | | | | – | | | | **–** | | | |
| Bradley 2016 | 50/28 | | | | CTCAE v4.0 | | | | | <1 | | | | | 18 | | | | | 0 | | | | | ***0*** | | | | | – | | | | | **–** | | | | | – | | | | | **–** | | | | | 1 | | | | ***6*** | | | |
| *Scanning* | | | | | | | | | | | | | | | | | | | | | | | | | | | | | | | | | | | | | | | | | | | | | | | | | | | | | | | | | |
| Garda 2022 | 50/25 | | | | CTCAE v4.0 | | | | | 0 | | | | | 11 | | | | | 0 | | | | | ***0*** | | | | | 0 | | | | | ***0*** | | | | | 1 | | | | | ***9*** | | | | | – | | | | **–** | | | |
| Salari 2021 | 43 or 50/16 or 25 | | | | – | | | | | 2 | | | | | 31 | | | | | – | | | | | **–** | | | | | – | | | | | **–** | | | | | – | | | | | **–** | | | | | 6 | | | | ***19*** | | | |
| Loap 2021 | 50/25 | | | | – | | | | | – | | | | | 1 | | | | | – | | | | | **–** | | | | | – | | | | | **–** | | | | | 1 | | | | | ***100*** | | | | | – | | | | **–** | | | |
| Mullikin 2020 | 50/25 | | | | – | | | | | – | | | | | 126 | | | | | – | | | | | **–** | | | | | 0 | | | | | ***0*** | | | | | – | | | | | **–** | | | | | – | | | | **–** | | | |
| Fattahi 2022 | 50/25 | | | | CTCAE v5.0 | | | | | – | | | | | 19 | | | | | – | | | | | **–** | | | | | – | | | | | **–** | | | | | 2 | | | | | ***11*** | | | | | – | | | | **–** | | | |
| Luo 2019 | 50/– | | | | CTCAE v4.0 | | | | | <3 | | | | | 42 | | | | | 0 | | | | | ***0*** | | | | | 10 | | | | | ***24*** | | | | | 5 | | | | | ***12*** | | | | | – | | | | **–** | | | |
| Zheng 2017 | 45-50/25-28 | | | | – | | | | | <6 | | | | | 100 | | | | | 0 | | | | | ***0*** | | | | | 0 | | | | | ***0*** | | | | | 1 | | | | | ***1*** | | | | | – | | | | **–** | | | |
| Verma 2017 | 50/25-28 | | | | CTCAE v4.0 | | | | | – | | | | | 93 | | | | | 1 | | | | | ***1*** | | | | | 27 | | | | | ***29*** | | | | | 47 | | | | | ***51*** | | | | | – | | | | **–** | | | |
| **Total** |  | | | |  | | | | |  | | | | | **469** | | | | | 1 | | | | | ***0*** | | | | | 46 | | | | | ***12*** | | | | | 57 | | | | | ***21*** | | | | | 7 | | | | ***14*** | | | |
| *Continued on next page.* | | | | | | | | | | | | | | | | | | | | | | | | | | | | | | | | | | | | | | | | | | | | | | | | | | | | | | | | | |
| **Table A6. Adverse outcomes after proton beam therapy to the whole breast or chest wall +/- regional lymph nodes in studies published 2000-2022 according to proton beam therapy type *(continued)*** | | | | | | | | | | | | | | | | | | | | | | | | | | | | | | | | | | | | | | | | | | | | | | | | | | | | | | | | | |
| **Author and year of publication** | | | | **PBT dose(Gy)/ No. fractions** | | | | **Grading system** | | | | | **Time assessed post PBT (months)** | | | | | **Total no. assessed** | | | | | **Severe** | | | | | | | | | | **Moderate** | | | | | | | | | | **Mild** | | | | | | | | | | **Unspecified** | | | | |
|  |  |  |  |  |  |  |  |  |  |  |  |  |  |  |  |  |  |  |  |  |  |  | **No. affected** | | | | | ***%*** | | | | | **No. affected** | | | | | ***%*** | | | | | **No. affected** | | | | | ***%*** | | | | | **No. affected** | | | | ***%*** |
| **Pneumonitis‡** | | | | | | | | | | | | | | | | | | | | | | | | | | | | | | | | | | | | | | | | | | | | | | | | | | | | | | | | | |
| *Scattering* | | | | | | | | | | | | | | | | | | | | | | | | | | | | | | | | | | | | | | | | | | | | | | | | | | | | | | | | | |
| Bradley 2016 | 50/28 | | | | CTCAE v4.0 | | | | | 3 | | | | | 18 | | | | | 0 | | | | | ***0*** | | | | | 2 | | | | | ***11*** | | | | | – | | | | | **–** | | | | | – | | | | **–** | | | |
| *Scattering or scanning* | | | | | | | | | | | | | | | | | | | | | | | | | | | | | | | | | | | | | | | | | | | | | | | | | | | | | | | | | |
| Jimenez 2019 | 45-50/25-28 | | | | CTCAE v4.0 | | | | | 4 | | | | | 69 | | | | | 0 | | | | | ***0*** | | | | | 1 | | | | | ***1*** | | | | | 3 | | | | | ***4*** | | | | | – | | | | **–** | | | |
| *Scanning* | | | | | | | | | | | | | | | | | | | | | | | | | | | | | | | | | | | | | | | | | | | | | | | | | | | | | | | | | |
| Garda 2022 | 50/25 | | | | CTCAE v4.0 | | | | | 32 | | | | | 11 | | | | | 0 | | | | | ***0*** | | | | | 0 | | | | | ***0*** | | | | | 0 | | | | | ***0*** | | | | | – | | | | **–** | | | |
| Loap 2021 | 50/25 | | | | – | | | | |  | | | | | 1 | | | | | 0 | | | | | ***0*** | | | | | 0 | | | | | ***0*** | | | | | 0 | | | | | ***0*** | | | | | – | | | | **–** | | | |
| Mullikin 2020 | 50/25 | | | | CTCAE v5.0 | | | | | – | | | | | 126 | | | | | 0 | | | | | ***0*** | | | | | 0 | | | | | ***0*** | | | | | 0 | | | | | ***0*** | | | | | – | | | | **–** | | | |
| Fattahi 2022 | 50/25 | | | | CTCAE v5.0 | | | | | – | | | | | 19 | | | | | – | | | | | **–** | | | | | – | | | | | **–** | | | | | 2 | | | | | ***11*** | | | | | – | | | | **–** | | | |
| Smith 2019 | 50/25 | | | | CTCAE v4.0 | | | | | – | | | | | 51 | | | | | 0 | | | | | ***0*** | | | | | 0 | | | | | ***0*** | | | | | 0 | | | | | ***0*** | | | | | – | | | | **–** | | | |
| Luo 2019 | 50/– | | | | CTCAE v4.0 | | | | | 12 | | | | | 42 | | | | | 1 | | | | | ***2*** | | | | | 0 | | | | | ***0*** | | | | | 0 | | | | | ***0*** | | | | | – | | | | **–** | | | |
| Cuaron 2017 | 49/– | | | | CTCAE v4.0 | | | | | – | | | | | 26 | | | | | 0 | | | | | ***0*** | | | | | 0 | | | | | ***0*** | | | | | 0 | | | | | ***0*** | | | | | – | | | | **–** | | | |
| **Total** |  | | | |  | | | | |  | | | | | **363** | | | | | 1 | | | | | ***0*** | | | | | 3 | | | | | ***1*** | | | | | 5 | | | | | ***1*** | | | | | – | | | | **–** | | | |
| **Rib fractures** | | | | | | | | | | | | | | | | | | | | | | | | | | | | | | | | | | | | | | | | | | | | | | | | | | | | | | | | | |
| *Scattering* | | | | | | | | | | | | | | | | | | | | | | | | | | | | | | | | | | | | | | | | | | | | | | | | | | | | | | | | | |
| Bradley 2016 | 50/28 | | | | CTCAE v4.0 | | | | | 7 | | | | | 18 | | | | | – | | | | | **–** | | | | | – | | | | | **–** | | | | | – | | | | | **–** | | | | | 1 | | | | ***6*** | | | |
| *Scattering or scanning* | | | | | | | | | | | | | | | | | | | | | | | | | | | | | | | | | | | | | | | | | | | | | | | | | | | | | | | | | |
| Jimenez 2019 | 45-50/25-28 | | | | CTCAE v4.0 | | | | | 16 | | | | | 69 | | | | | 0 | | | | | ***0*** | | | | | 0 | | | | | ***0*** | | | | | 5 | | | | | ***7*** | | | | | – | | | | **–** | | | |
| *Scanning* | | | | | | | | | | | | | | | | | | | | | | | | | | | | | | | | | | | | | | | | | | | | | | | | | | | | | | | | | |
| Garda 2022 | 50/25 | | | | CTCAE v4.0 | | | | | 32 | | | | | 11 | | | | | 0 | | | | | ***0*** | | | | | 0 | | | | | ***0*** | | | | | 0 | | | | | ***0*** | | | | | – | | | | **–** | | | |
| Mullikin 2020 | 50/25 | | | | CTCAE v5.0 | | | | | 14 | | | | | 126 | | | | | 0 | | | | | ***0*** | | | | | 0 | | | | | ***0*** | | | | | 1 | | | | | ***1*** | | | | | – | | | | **–** | | | |
| Fattahi 2022 | 50/25 | | | | – | | | | | 44 | | | | | 19 | | | | | – | | | | | **–** | | | | | – | | | | | **–** | | | | | – | | | | | **–** | | | | | 4 | | | | ***21*** | | | |
| Smith 2019 | 50/25 | | | | – | | | | | – | | | | | 51 | | | | | – | | | | | **–** | | | | | – | | | | | **–** | | | | | – | | | | | **–** | | | | | 0 | | | | ***0*** | | | |
| Luo 2019 | 50/– | | | | CTCAE v4.0 | | | | | >3 | | | | | 42 | | | | | 0 | | | | | ***0*** | | | | | 0 | | | | | ***0*** | | | | | 0 | | | | | ***0*** | | | | | 0 | | | | ***0*** | | | |
| Verma 2017 | 50/25-28 | | | | CTCAE v4.0 | | | | | 39 | | | | | 91 | | | | | – | | | | | **–** | | | | | – | | | | | **–** | | | | | – | | | | | **–** | | | | | 2 | | | | ***2*** | | | |
| Cuaron 2017 | 49/– | | | | CTCAE v4.0 | | | | | – | | | | | 26 | | | | | 0 | | | | | ***0*** | | | | | 0 | | | | | ***0*** | | | | | 0 | | | | | ***0*** | | | | | – | | | | **–** | | | |
| **Total** |  | | | |  | | | | |  | | | | | **453** | | | | | 0 | | | | | ***0*** | | | | | 0 | | | | | ***0*** | | | | | 6 | | | | | ***2*** | | | | | 7 | | | | ***3*** | | | |
| *Continued on next page.* | | | | | | | | | | | | | | | | | | | | | | | | | | | | | | | | | | | | | | | | | | | | | | | | | | | | | | | | | |
| **Table A6. Adverse outcomes after proton beam therapy to the whole breast or chest wall +/- regional lymph nodes in studies published 2000-2022 according to proton beam therapy type *(continued)*** | | | | | | | | | | | | | | | | | | | | | | | | | | | | | | | | | | | | | | | | | | | | | | | | | | | | | | | | | |
| **Author and year of publication** | | | | **PBT dose(Gy)/ No. fractions** | | | | | **Grading system** | | | | | **Time assessed post PBT (months)** | | | | | **Total no. assessed** | | | | | **Severe** | | | | | | | | | | **Moderate** | | | | | | | | | | **Mild** | | | | | | | | | **Unspecified** | | | | |
|  |  |  |  |  |  |  |  |  |  |  |  |  |  |  |  |  |  |  |  |  |  |  |  | **No. affected** | | | | | ***%*** | | | | | **No. affected** | | | | | ***%*** | | | | | **No. affected** | | | | | ***%*** | | | | **No. affected** | | | | ***%*** |
| **Telangiectasia** | | | | | | | | | | | | | | | | | | | | | | | | | | | | | | | | | | | | | | | | | | | | | | | | | | | | | | | | | |
| *Scattering or scanning* | | | | | | | | | | | | | | | | | | | | | | | | | | | | | | | | | | | | | | | | | | | | | | | | | | | | | | | | | |
| Jimenez 2019 | 45-50/25-28 | | | | CTCAE v4.0 | | | | | 12 | | | | | 69 | | | | | – | | | | | **–** | | | | | – | | | | | **–** | | | | | 11 | | | | | ***16*** | | | | | *–* | | | | **–** | | | |
| *Scanning* | | | | | | | | | | | | | | | | | | | | | | | | | | | | | | | | | | | | | | | | | | | | | | | | | | | | | | | | | |
| Mullikin 2020 | 50/25 | | | | CTCAE v5.0 | | | | | – | | | | | 126 | | | | | 0 | | | | | ***0*** | | | | | 0 | | | | | ***0*** | | | | | 1 | | | | | ***1*** | | | | | *–* | | | | **–** | | | |
| Fattahi 2022 | 50/25 | | | | CTCAE v5.0 | | | | | 0 | | | | | 13 | | | | | 0 | | | | | ***0*** | | | | | 0 | | | | | ***0*** | | | | | 0 | | | | | ***0*** | | | | | 0 | | | | ***0*** | | | |
| Luo 2019 | 50/– | | | | CTCAE v4.0 | | | | | >3 | | | | | 42 | | | | | – | | | | | **–** | | | | | 0 | | | | | ***0*** | | | | | 1 | | | | | ***2*** | | | | | *–* | | | | **–** | | | |
| Cuaron 2017 | 49/– | | | | CTCAE v4.0 | | | | | – | | | | | 26 | | | | | – | | | | | **–** | | | | | – | | | | | **–** | | | | | 1 | | | | | ***4*** | | | | | *–* | | | | **–** | | | |
| **Total** |  | | | |  | | | | |  | | | | | **276** | | | | | **0** | | | | | ***0*** | | | | | **0** | | | | | ***0*** | | | | | **14** | | | | | ***5*** | | | | | **0** | | | | ***0*** | | | |

**Footnotes**

Denominators for each study vary by adverse outcome as not all studies assessed all patients at all timepoints for all adverse outcomes.

*Includes reports of pain from skin, breast and unspecified pain.

†Oesophagitis includes reports of dysphagia.

‡Pneumonitis includes reports of cough and dyspnoea.

§Fibrosis includes reports of induration.

Abbreviations: CTCAE=Common Terminology Criteria for Adverse Events, Gy=Gray, No.=number, PBT=proton beam therapy, v=version. Blank or "–"=not specified

# **Figure A2. Meta-analyses of the percentage of patients with moderate and mild dermatitis after scanning proton beam therapy to the whole breast or chest wall +/- regional lymph nodes.** The estimates for the total percentages and confidence intervals allow for heterogeneity between studies.

# **
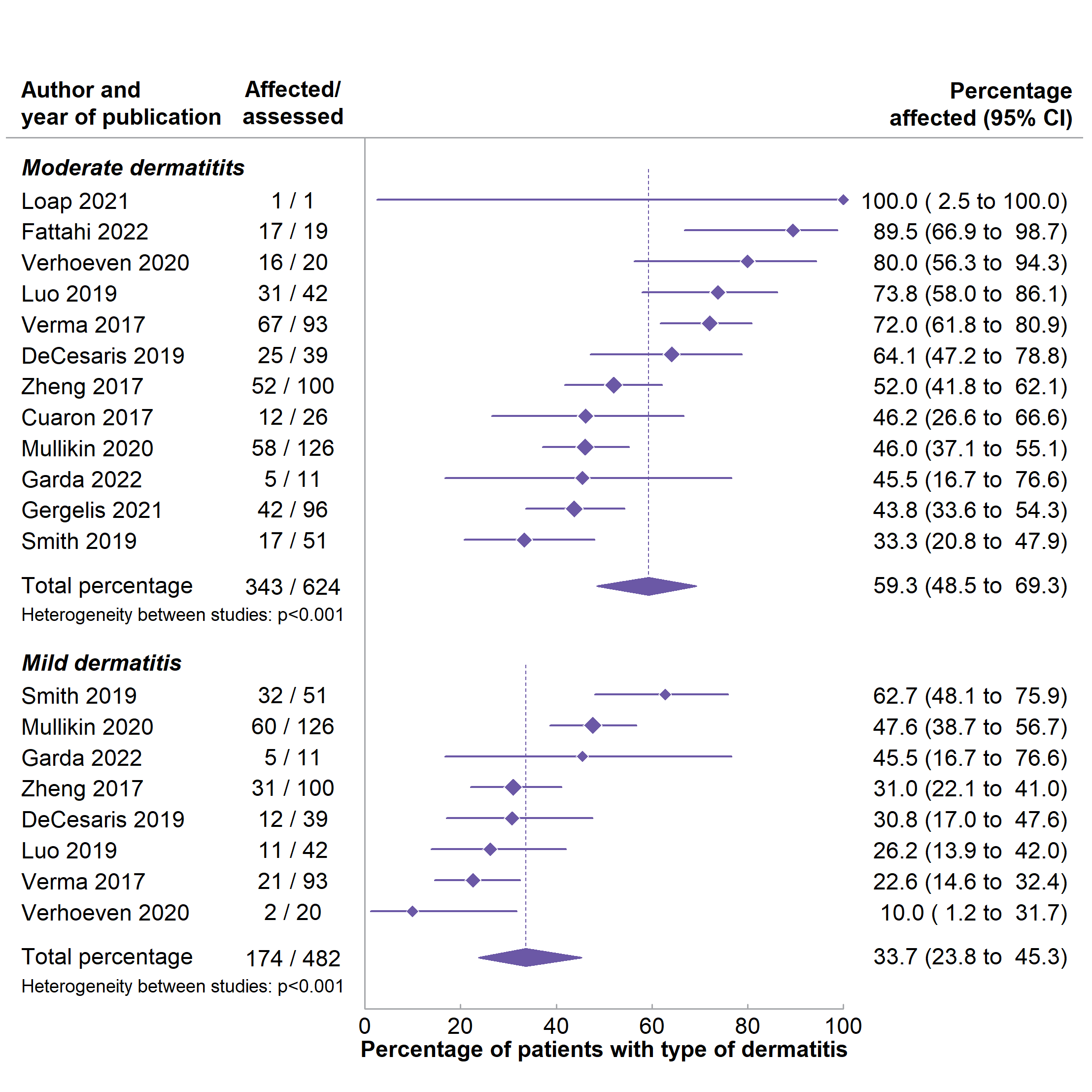
**

| **Table A7a. Ongoing randomised studies of proton beam therapy for early breast cancer** | | | | | | | | | | | | | | |  |
| --- | --- | --- | --- | --- | --- | --- | --- | --- | --- | --- | --- | --- | --- | --- | --- |
| **Study ID Country** | **Estimated study years** | | **Estimated no. patients** | | **Study arms: Intervention** | | | | |  | **Planned study outcomes** | | | |  |
|  |  |  |  |  | **Radiotherapy type** | | **Total dose (Gy)/ No. fractions** | | |  | **Primary** | | **Secondary** | |  |
| **Whole breast/chest wall +/- regional lymph nodes (3298 patients)** | | | | | | | | | | | | | | |  |
| **NCT04291378** Denmark | 2020-37 | | 1502 | | PBT | | 50/25 | | |  | Ischaemic and valvular heart disease at 10 years | | Second cancers, distant failure, acute and late morbidity, patient reported outcome measures, incidence of cardiac disease on blood markers and imaging | |  |
|  |  |  |  |  | Photon | | 50/25 | | |  |  |  |  |  |  |
| **NCT02603341*** USA | 2016-32 | | 1278 | | PBT | | 50/25  (+/- SIB) | | |  | Major cardiovascular events at 10 years | | Disease control, quality of life, association between radiation dose distribution to heart and other normal tissues with quality of life and cardiac toxicity, breast cancer specific survival, overall survival | |  |
|  |  |  |  |  | Photon | | 50/25 (+/- SIB) | | |  |  |  |  |  |  |
| **ISRCTN14220944** UK | 2022-30 | | 192 | | PBT: scanning | | 40/15 | | |  | Mean heart dose, and patient-reported normal tissue toxicity in the breast at 2 years | | Lung and contralateral breast doses, clinician-reported early and late toxicity at 1 year, health-related quality of life, health economic consequences, changes to planned RT pathway, second cancers at 5 years, recurrence and survival at 5 years, incidence of major cardiac events at 5 years, other dosimetric outcomes | |  |
|  |  |  |  |  | Photon: IMRT | | 40/15 | | |  |  |  |  |  |  |
| **TCTR20210219004** Thailand | 2021 - 21 | | 140 | | PBT | | "Ultrahypofractionation" | | |  | Acute skin toxicity at 3 months | | Cosmesis at 3 months, late toxicity at 1 year, locoregional recurrence and distant metastases at 2 years | |  |
|  |  |  |  |  | Photon | | "Ultrahypofractionation" | | |  |  |  |  |  |  |
| **NCT04443413** USA | 2020-23 | | 98 | | PBT | | –/5  (+/- 5 boost) | | |  | ≥ grade 3 adverse events, unplanned surgical intervention after reconstruction at 2 years | | Acute and late toxicity, 5 year locoregional control, disease free survival, cause-specific survival, overall survival | |  |
|  |  |  |  |  | Photon | | –/25  (+/- 4 boost) | | |  |  |  |  |  |  |
| **NCT02783690** USA | 2016-25 | | 88 | | PBT: scanning | | 40/15 | | |  | ≥ grade 3 adverse events, unplanned surgical intervention after reconstruction at 2 years | | Acute and late adverse events, quality of life, cosmesis, ipsilateral breast tumour recurrence, distant recurrence, disease free survival, overall survival | |  |
|  |  |  |  |  | PBT: scanning | | 50/25 | | |  |  |  |  |  |  |
| **Footnotes as for Table A7b** | | | | | | | | | | | | | | |  |
| **Table A7b. Ongoing non-randomised studies of proton beam therapy for early breast cancer** | | | | | | | | | | | | | | | |
| **Study ID Country** | | **Estimated study years** | | **Estimated no. patients** | | **Study arms: Intervention** | | |  | | | **Planned study outcomes** | | | |
|  |  |  |  |  |  | **Radiotherapy type** | | **Total dose (Gy)/ No. fractions** |  | | | **Primary** | | **Secondary** | |
| **Whole breast/chest wall +/- regional lymph nodes (285 patients)** | | | | | | | | | | | | | | | |
| **NCT01758445** USA | | 2013-30 | | 220 | | PBT | | – /≥28  (+/- 6-9) |  | | | Acute and late toxicites at 5 years | | Local control, regional control, disease free survival, second cancers, cardiac disease, quality of life, dosimetry | |
| **NCT02725840** USA | | 2016-22 | | 41 | | PBT | | – |  | | | Lung vascular damage on CT at 2 years; blood cytokines post radiation at 2 years; correlations between vascular damage on CT scans with pulmonary function test outcomes | | ≥Grade 2 lung toxicity, pattern of lung metastases, overall survival | |
|  |  |  |  |  |  | Photon: IMRT | | – |  | | |  |  |  |  |
| **NCT02199366** USA | | 2014-21 | | 24 | | PBT | | – |  | | | Changes in cardiac function on cardiac MRI at baseline and 1 year | | Major cardiac side effects (myocardial infarction, valve disorder, congestive heart failure, and angina), quality of life | |
|  |  |  |  |  |  | Photon | | – |  | | |  |  |  |  |
| *Continued on next page.* | | | | | | | | | | | | | | | |
| **Table A7b. Ongoing non-randomised studies of proton beam therapy for early breast cancer *(continued)*** | | | | | | | | | | | | | | | |
| **Study ID Country** | | **Estimated study years** | | **Estimated no. patients** | | **Study arms: Intervention** | | |  | | | **Planned study outcomes** | | | |
|  |  |  |  |  |  | **Radiotherapy type** | | **Total dose (Gy)/ No. fractions** |  | | | **Primary** | | **Secondary** | |
| **Partial breast (765 patients)** | | | | | | | | | | | | | | | |
| **NCT01245712** USA | | 2010-22 | | 200 | | PBT | | – /10 |  | | | Patient reported cosmesis at 1 year | | Local control, acute ≥grade 3 dermatitis | |
| **NCT02453737** USA | | 2015-22 | | 198 | | PBT | | 21.9/3 |  | | | Change in rate of fair/poor cosmesis at 3 years compared with baseline | | – | |
|  |  |  |  |  |  | Photon: 3DCRT | | 21.9/3 |  | | |  |  |  |  |
|  |  |  |  |  |  | Brachytherapy | | 21.0/3 |  | | |  |  |  |  |
| **NCT01310530** USA | | 2011-25 | | 150 | | PBT | | 40/10 |  | | | Breast cancer recurrence at 5 years | | Breast cancer recurrence in low versus intermediate risk women, disease free survival, treatment side-effects | |
| **NCT01766297*** USA | | 2013-33 | | 132 | | PBT | | 40/10 |  | | | Freedom from ipsilateral breast tumour recurrence at 3 years | | Acute and late toxicity, dosimetry, quality of life, recurrence pattern, overall survival | |
| **NCT01839838** USA | | 2013-24 | | 57 | | PBT | | – |  | | | Adverse events at 5 years | | – | |
| **NCT00599989** USA | | 2005-09 | | 28 | | Photon: 3DCRT | | – |  | | | Acute toxicity at 1 week, 4 weeks and 3 months | | Site of disease recurrence, time to recurrence, late toxicity, cosmesis | |
|  |  |  |  |  |  | Interstitial brachytherapy | | – |  | | |  |  |  |  |
|  |  |  |  |  |  | Intracavity balloon brachytherapy | | – |  | | |  |  |  |  |
|  |  |  |  |  |  | PBT | | – /10 |  | | |  |  |  |  |
| *Continued on next page.* | | | | | | | | | | | | | | | |
| **Table A7b. Ongoing non-randomised studies of proton beam therapy for early breast cancer *(continued)*** | | | | | | | | | | | | | | | |
| **Study ID Country** | | **Estimated study years** | | **Estimated no. patients** | | **Study arms: Intervention** | | |  | | | **Planned study outcomes** | | | |
|  |  |  |  |  |  | **Radiotherapy type** | | **Total dose (Gy)/ No. fractions** |  | | | **Primary** | | **Secondary** | |
| **Clinical target not specified (5443 patients)** | | | | | | | | | | | | | | | |
| **NCT00991094** USA | | 2005-22 | | 5000† | | PBT | | – |  | | | Acute and late toxicities | | Symptom burden during 3 months post treatment | |
| **ChiCTR2000031985** China | | 2020-35 | | 299 | | PBT | | – |  | | | Heart injury | | – | |
| **CTRI/2020/11/029415** India | | 2020-25 | | 100 | | PBT | | – |  | | | Rate of acute coronary events over 5 years | | Locoregional control, disease free survival, overall survival, changes in cardiac function, cosmesis, quality of life over 5 years | |
| **JPRN-UMIN 000017579** Japan | | 2015-25 | | 24 | | PBT | | 62/26 |  | | | Adverse events of normal tissue | | Location of local recurrence, duration of distant recurrence, overall survival, cosmesis | |
| **JPRN-UMIN 000016206** Japan | | 2015- | | 20 | | PBT | | 60-72/10 |  | | | Local control Acute toxicity | | – | |

**Footnotes**

*Some patients enrolled in this randomised trial are also enrolled in ancillary observational studies: NCT04361240 study including 155 participants planned for 2020-23; NCT03270072 study including 100 participants planned for 2017-27.

†Includes patients with other cancers treated with proton beam therapy at University of Texas M.D. Anderson Cancer Center.

| Abbreviations: CT=computed tomography; IMRT=Intensity Modulated Radiotherapy; MRI=magnetic resonance imaging; No.=number; PBT=proton beam therapy; 3DCRT=Three-dimensional conformal external-beam photon radiotherapy; SIB=simultaneous integrated boost, "–"=not specified |
| --- |
